# A systems genetics approach reveals PbrNSC as a regulator of lignin and cellulose biosynthesis in stone cells of pear fruit

**DOI:** 10.1186/s13059-021-02531-8

**Published:** 2021-11-14

**Authors:** Runze Wang, Yongsong Xue, Jing Fan, Jia-Long Yao, Mengfan Qin, Tao Lin, Qun Lian, Mingyue Zhang, Xiaolong Li, Jiaming Li, Manyi Sun, Bobo Song, Jiaying Zhang, Kejiao Zhao, Xu Chen, Hongju Hu, Zhangjun Fei, Cheng Xue, Jun Wu

**Affiliations:** 1 College of Horticulture, State Key Laboratory of Crop Genetics and Germplasm Enhancement, Nanjing Agricultural University, Nanjing, 210095 China; 2 Institute of Fruit and Tea, Hubei Academy of Agricultural Sciences, Wuhan, 430072 China; 3 The New Zealand Institute for Plant & Food Research Limited, Auckland, 1025 New Zealand; 4 Genome Analysis Laboratory of the Ministry of Agriculture, Agricultural Genomics Institute at Shenzhen, Chinese Academy of Agricultural Sciences, Shenzhen, 518124 China; 5 College of Horticulture, China Agricultural University, Beijing, 100083 China; 6 State Key Laboratory of Crop Biology, College of Horticulture Science and Engineering, Shandong Agricultural University, Tai’an, 271018 China; 7 Haixia Institute of Science and Technology, Horticultural Plant Biology and Metabolomics Center, Fujian Agriculture and Forestry University, Fuzhou, 350002 China; 8 Boyce Thompson Institute, Cornell University, Ithaca, NY 14853 USA; 9 USDA-ARS, Robert W. Holley Center for Agriculture and Health, Ithaca, NY 14853 USA

**Keywords:** Pear fruit, Co-expression network, eQTL, Stone cell formation, NAC transcription factor, Transcriptional regulation

## Abstract

**Background:**

Stone cells in fruits of pear (*Pyrus pyrifolia*) negatively influence fruit quality because their lignified cell walls impart a coarse and granular texture to the fruit flesh.

**Results:**

We generate RNA-seq data from the developing fruits of 206 pear cultivars with a wide range of stone cell contents and use a systems genetics approach to integrate co-expression networks and expression quantitative trait loci (eQTLs) to characterize the regulatory mechanisms controlling lignocellulose formation in the stone cells of pear fruits. Our data with a total of 35,897 expressed genes and 974,404 SNPs support the identification of seven stone cell formation modules and the detection of 139,515 eQTLs for 3229 genes in these modules. Focusing on regulatory factors and using a co-expression network comprising 39 structural genes, we identify PbrNSC as a candidate regulator of stone cell formation. We then verify the function of *PbrNSC* in regulating lignocellulose formation using both pear fruit and *Arabidopsis* plants and further show that PbrNSC can transcriptionally activate multiple target genes involved in secondary cell wall formation.

**Conclusions:**

This study generates a large resource for studying stone cell formation and provides insights into gene regulatory networks controlling the formation of stone cell and lignocellulose.

**Supplementary Information:**

The online version contains supplementary material available at 10.1186/s13059-021-02531-8.

## Background

Pear (*Pyrus pyrifolia*) belongs to the Rosaceae family and is one of the most economically important temperate tree fruit crops [1], with an annual worldwide production of ~ 24 million tons (2019, FAOSTAT). Different from other Rosaceae species, pear accumulates a large number of stone cells in the flesh of its fruits. These cell clusters with thickened cell wall materials cause a gritty texture and a poor fruit taste and negatively impact on consumer satisfaction. Stone cells are a type of sclerenchyma cells, in which secondary cell walls are deposited on the primary walls after cessation of cell expansion [2, 3]. Lignin and cellulose, two of the most abundant biopolymers on earth [4–6], are the main components of the secondary cell wall of stone cells [7, 8]. The formation of stone cells is known to be closely related to the biosynthesis, transfer, and deposition of lignin and cellulose in pear fruit flesh [8–12].

Understanding the genetic regulation of lignin and cellulose synthesis and accumulation in fruit is of great value for the improvement of fruit quality. The pear *PbrMYB169* gene has been reported to be implicated in lignification. It activates the expression of lignin biosynthesis genes during stone cell development in pear fruit [13]. In loquat, the transcription factors EjMYB1, EjNAC1, EjAP2-1, and EjHSF3 regulate lignification in cold-injured fruits by activating lignin biosynthesis genes [14–17]. These studies have provided an excellent starting point for understanding the process of fruit lignification. However, our understanding of the gene regulatory networks of lignin and cellulose accumulation in fruit tissues remains limited. With rapid advances of sequencing technologies and significant decreases in costs, increasing reports of genome and transcriptome analyses have provided large amounts of genome-wide information and deepened our understanding of important fruit traits. Recently, we reported genome-wide association studies (GWAS) on eleven pear fruit traits, including stone cell contents, and identified one gene, *PbrSTONE*, that has been functionally verified to be involved in stone cell formation [18]. However, fruit-related traits usually have complex gene expression and regulation mechanisms [19]. The RNA sequencing (RNA-seq) can provide abundant gene expression variation among pear individuals, deepening our understanding of underlying regulatory networks of fruit traits [20]. Using population-scale RNA-seq, genetic regulation of complex trait can be explored through combining gene co-expression and expression quantitative trait locus (eQTL) analyses, an approach called systems genetics [21, 22]. The eQTL approach has been demonstrated in plants including *Arabidopsis*, maize, and tomato [23–25], but systems genetics deciphering of complex agronomic traits remains limited.

In this study, we analyzed RNA-seq data generated from fruit flesh samples collected at 49 days after full bloom (DAFB) from 206 sand pear cultivars with varying contents of stone cells. We generated gene co-expression networks for pear lignin and cellulose biosynthesis and performed an eQTL analysis which identified SNP-gene associations. Systems genetics through integrating gene co-expression networks and eQTLs identified a candidate NAC gene named as NAC STONE CELL PROMOTING FACTOR (*PbrNSC*) that is potentially involved in the regulation of lignin and cellulose biosynthesis in pear fruit. Moreover, PbrNSC was verified to regulate stone cell secondary cell wall (SCW) formation during pear fruit development and its LP- and WQ-box motifs are responsible for its transcriptional activation function. This study contributes to our understanding of gene regulatory networks of lignocellulose formation in stone cells of pear fruit and can inform efforts to increase the consumption value of pears.

## Results

### Phenotypic determination and global gene expression profiling of pear fruit

**Fig. 1:**
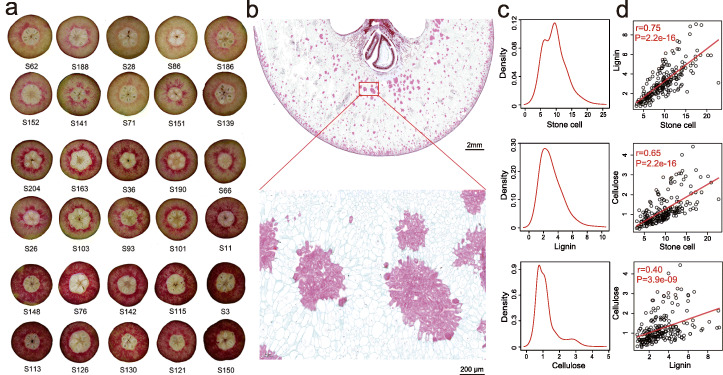
Overview of the three phenotypic traits assessed in this study. **a** Fruits of 30 representative pear cultivars were cross-sectioned at 49 days after full bloom (DAFB) and stained with phloroglucinol-HCl to show the red-stained stone cells. **b** Distribution and appearance of stone cells in fruit flesh analyzed at 49 DAFB by light microscopy. **c** Distribution of stone cell contents, and lignin and cellulose levels in stone cell. The *x*-axis indicates the contents (g/100 g fruit flesh fresh weight), and the *y*-axis shows the probability density estimated by kernel density estimation (KDE). **d** Pairwise correlation between the contents (g/100 g fruit flesh fresh weight) of stone cells, lignin, and cellulose in 206 pear cultivars

We collected fruit flesh samples at 49 DAFB from a total of 206 sand pear cultivars (*P. pyrifolia*) that collectively represented a broad scope of genetic diversity and large phenotypic variability (Additional file 1). The fruits of these pear cultivars had varied contents of stone cells (Fig. 1a, b), which varied from 3.2 to 22.6 (g/100 g), over a 7-fold range, among the 206 cultivars tested. Given that the main components of the secondary walls of stone cells are lignin and cellulose, we also measured the contents of lignin and cellulose in stone cells (Fig. 1c) and found a positive correlation between lignin, cellulose, and stone cell contents in sand pear cultivars (Fig. 1d).

Global gene expression profiling using RNA-seq was also carried out on above flesh fruit samples. An average of more than 53 million high-quality reads (~ 8 Gb) were obtained for each sample (Additional file 1), which were aligned to the “Dangshansuli” pear genome, with an average mapping rate of 72.6%. Among genes mapped with RNA-seq reads, an average of 84.5% had more than 50% of the CDS length covered by RNA-seq reads (Additional file 2: Fig. S1a; Additional file 3). The expression of a total of 35,897 genes was detected in the RNA-seq dataset, accounting for 84.9% of the annotated genes in the pear genome. An average of 25,636 expressed genes were detected in each pear cultivar (Additional file 1).

### Identification of stone cell formation modules

**Fig. 2:**
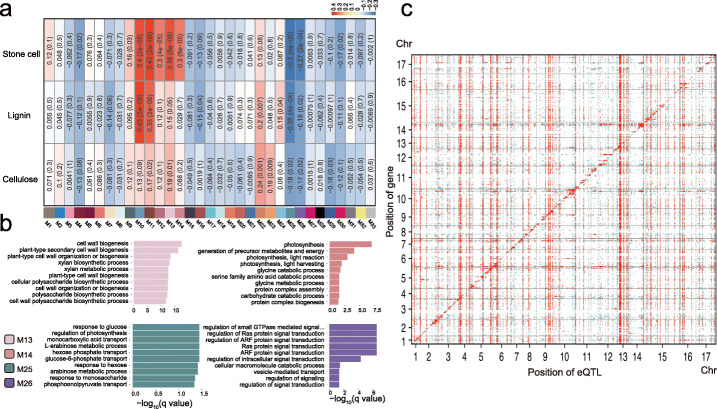
Gene co-expression modules and eQTL mapping. **a** Heat map of the correlations between the detected modules (M1 to M33) and stone cell, lignin, and cellulose contents. Numbers within the heatmap represent correlation coefficients (*r* values) and *P* values (in parentheses). The color scale at the right-top corner indicates module-trait *r* values. **b** GO enrichment analysis for M13, M14, M25, and M26. The top 10 enriched GO terms are shown. **c** Genomic distribution of eQTLs identified in stone cell formation modules in the pear fruit flesh. The *x*-axis indicates the physical positions of eQTLs, and the *y*-axis shows the physical positions of expressed genes. The gray lines separate the 17 pear chromosomes. The *P* value of each eQTL was analyzed using a Bonferroni test and is represented by three spot colors in the plot, red for the top 20%, blue for the bottom 20%, and gray for the rest

WGCNA [26] was performed to identify candidate trait-linked modules based on the expression profiles of 22,842 genes in the fruit flesh of the 206 cultivars. A total of 33 modules consisting of 21,804 genes were identified (M1-M33) (Fig. 2a), while the remaining 1038 genes were considered outliers and excluded from further analysis. We then performed module-trait association studies [26] and identified seven modules comprising 4383 genes as potential modules involved in stone cell formation for their significant correlation with stone cell contents (M10-M14 and M25-M26) (*P ≤* 0.01) (Fig. 2a). The numbers of genes in these seven modules were 2219 (M14), 1518 (M11), 232 (M13), 140 (M12), 117 (M26), 77 (M25), and 80 (M10), respectively.

Gene Ontology (GO) analysis of genes in the seven stone cell formation modules showed many enrichment processes putatively related to secondary cell wall metabolism (Fig. 2b; Additional file 4). The detected enriched processes included cell wall biogenesis and xylan metabolic process (M13) and photosynthesis (M14). Interestingly, in addition to “secondary cell wall metabolism” terms, GO analysis also revealed enrichment for “response to glucose” (M25) and “auxin response factor (ARF) protein signal transduction” (M26). Cellulose is a giant polymer comprising glucose monomers, and secondary cell wall formation is known to be regulated by auxin signaling [27, 28]. We also noted that genes in modules M11 exhibited enrichment for annotations related to “response to oxygen-containing compound,” suggesting a potential functional contribution of oxygen-containing compound stimulus in stone cell formation.

### eQTLs of stone cell formation modules

A high-quality set of 974,404 SNPs (minor allele frequency > 5%, missing rate < 20%) was called from the RNA-seq data. In the SNP set, 97.1% of SNPs were located within 28,238 genes, with an average of 33.5 SNPs per gene (Additional file 2: Fig. S1b; Additional file 5). The phylogenetic tree constructed using SNPs at fourfold degenerate sites suggested two distinct clades with geographic separation feature, with Clade I containing accessions from China and Clade II containing accessions from Japan and Korea, which was also supported by the results of the population structure and the principal component analyses (Additional file 2: Fig. S2).

To determine the effect of genetic variants potentially involved in the regulation of expression, a total of 25,828 genes with a median expression level greater than zero among the cultivars were used for eQTL mapping. At a rigorous Bonferroni-corrected *α* = 0.05 (corresponding to *P* = 1.99 × 10^−12^), we detected a total of 320,633 SNPs significantly associated with 20,210 genes. To reduce the eQTL redundancy of certain genes, the leading SNP within a 20-kb interval was selected and defined as an eQTL according to a previous method [23] (Additional file 2: Fig. S3). We finally obtained 833,872 eQTLs putatively regulating the expression of 18,435 genes.

The eQTLs were further classified as local or distant according to their relative distance from their associated genes [23]. Among the mapped eQTLs, a total of 4602 local and 829,270 distant were identified for 4602 and 17,942 genes, respectively. Furthermore, a total of 111 distant eQTL hotspots (permutation test, *P* value ≤ 0.01; Additional file 6) were identified across the pear genome. Fruit sugars, organic acids, aroma compounds, and stone cells are all important components determining pear fruit quality. A total of 62 local and 12,310 distant eQTLs were identified for 62 and 277 structural genes, respectively, with annotated functions related to sugar, organic acid, aroma compound metabolisms (Additional files 7 and 8), and a total of 689 local and 138,826 distant eQTLs were identified for 689 and 3164 genes in stone cell modules, respectively (Fig. 2c; Additional files 9 and 10).

### Structural genes and co-expression network of the lignocellulose pathway

**Fig. 3:**
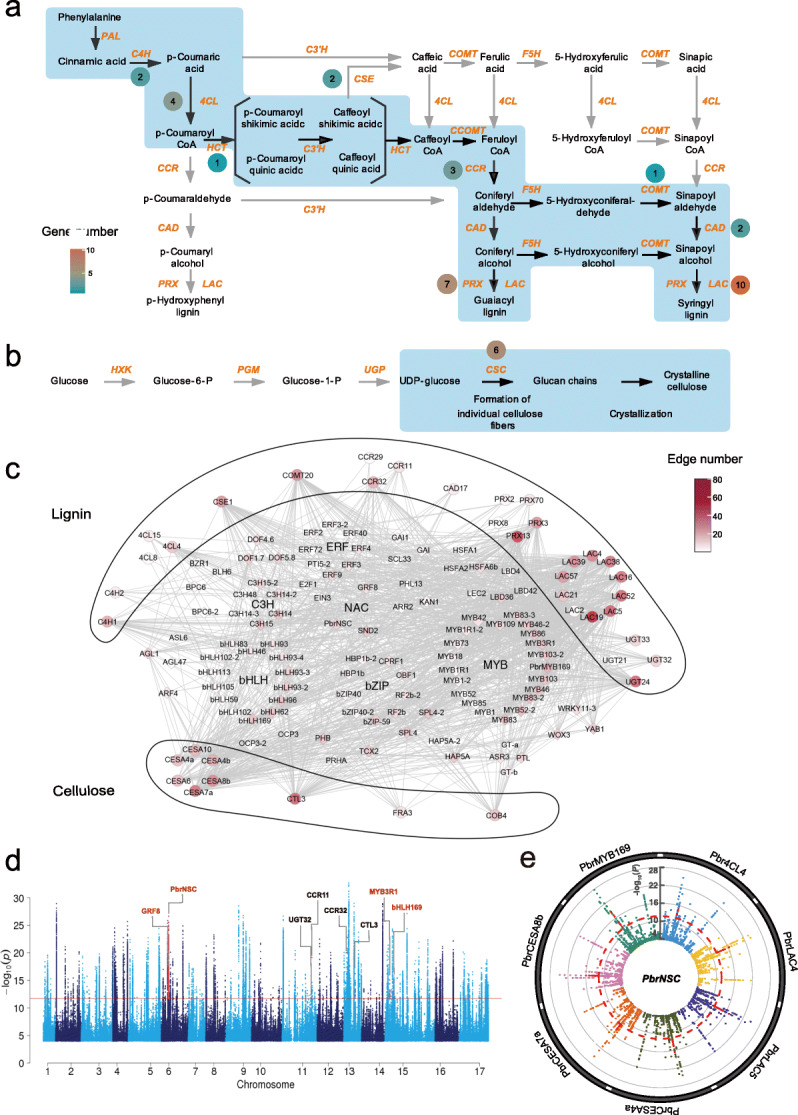
Co-expression network and candidate eQTLs associated with lignin and cellulose structural genes of pear. **a** Genes in the lignin biosynthesis pathway in pear, adapted from Humphreys and Chapple (2002) [29]. **b** Genes in the cellulose biosynthesis pathway in pear, adapted from Balaji et al. (2017) [30]. Black arrows and blue shadows indicate key reaction steps. Genes in orange indicate enzymes in the pathways. Round boxes next to the enzymes contain the numbers of core pathway genes. **c** Co-expression network of the lignin and cellulose biosynthesis. The outer ring of the network represents structural genes implicated in lignin and cellulose synthesis (circles), while inside are TFs (rhombus). Nodes are labeled with gene names colored based on the edge number. TFs and pathway genes are connected via undirected edges. Internal connections among TFs and among pathway genes were not shown for clarity. Detailed IDs, names, and connection information for all genes in the network can be found in Additional files 12 and 13. **d** Combined Manhattan plots of eQTLs of genes associated with lignin and cellulose biosynthesis. The red horizontal line depicts the significance threshold (*P* = 1.99 × 10^−12^). All significant SNP signals in local and distant eQTLs were combined. Genes in black indicate those located in local eQTLs, while genes in red indicate those located in distant eQTLs. A full list of gene names is provided in Additional file 12. **e** Circle Manhattan plot for chromosome 6 from the eQTL results of *Pbr4CL4*, *PbrLAC4*, *PbrLAC5*, *PbrCESA4a*, *PbrCESA7a*, *PbrCESA8b*, and *PbrMYB169*. The plot was constructed using the “CMplot” package in R software. The red circular dotted line depicts the significance threshold (*P* = 1.99 × 10^−12^). The signal points highlighted in red indicated the locus of *PbrNSC*

We identified a total of 491 lignin-related structural genes belonging to 14 families and 55 cellulose-related structural genes belonging to nine families in the pear genome (Additional file 11). Among them, 58 lignin- and cellulose-related structural genes were in stone cell modules. We then focused on these structural genes in stone cell modules and performed gene-trait association studies using the “corPvalueStudent” function of WGCNA, which identified a total of 45 structural genes that were significantly associated with stone cell contents in pear fruit, among which 36 were related to lignin and 9 to cellulose (*P* value < 0.05; Fig. 3a, b). Among the stone cell modules, the M13 module contains the highest number of lignin- and cellulose-related structural genes (18).

Moreover, we constructed a lignocellulose co-expression network including transcription factors (TFs) and the 39 lignin and cellulose structural genes based on gene expression profiles of the 206 pear cultivars and of 7 different developmental stages in three cultivars, while the remaining six structural genes were not co-expressed with any annotated TFs and thus excluded from further analysis (Fig. 3c; Additional files 12 and 13). In this network, in addition to that the structural genes were apparently closely related to NAC and MYB TF families which have been previously demonstrated to be involved in secondary cell wall regulation, we also found that the structural genes were connected to many other TFs, together constituting a dense co-expression network (Additional file 2: Fig. S4). Specifically, we found two NAC TFs in the lignocellulose co-expression network, and the NAC gene with the most connections was *SND2* (*Pbr000412.1*), whose homolog in *Populus* positively regulates fiber SCW thickening [31]. A total of eight hub MYB TFs (permutation test, *P* value ≤ 0.01) (Additional file 12) were found in the lignocellulose co-expression network, among which *MYB83* (*Pbr004921.1* and *Pbr023831.1*), *MYB52* (*Pbr028319.1*), *MYB46* (*Pbr020666.1*), *MYB103* (*Pbr038701.2*), and *PbrMYB169* (*Pbr012624.1*) showed a higher number of connections with structural genes. The homolog of *MYB83*, *MYB52*, *MYB46*, and *MYB103* in *Arabidopsis* has been reported to function in secondary cell wall formation [32–34], while *PbrMYB169* was previously reported to activate lignin biosynthesis genes during stone cell development in pear fruit [13].

The homologs of other genes in the network such as *bHLH62* (*Pbr014934.1*), *C3H14* (*Pbr032568.1* and *Pbr037100.1*), *BLH6* (*Pbr015799.1*), and *GAI* (*Pbr016671.1*) have also been reported to have secondary cell wall-related functions [33, 35–38]. In addition, our network also discovered many unreported genes (e.g., genes from G2-like, and SBP family) that may functionally impact stone cell formation in pear. These results highlight the complex regulatory nature of the lignin and cellulose pathways and indicate that many transcription factors are apparently engaged in the process of stone cell formation.

### PbrNSC is a potential regulator of the lignocellulose pathway

We integrated the lignocellulose co-expression network and stone cell eQTL map to identify potential regulators of the lignin and cellulose pathways. Of the aforementioned 39 structural genes in the lignocellulose co-expression network, our stone cell eQTL map showed that 36 had at least one eQTL. Among these 36 genes, 32 were regulated by distant eQTLs, whereas the remaining four were regulated by both distant and local eQTLs. We considered a gene as a candidate regulator if the gene located in a given eQTL region was co-expressed with the gene for which the eQTL was detected, and the corresponding eQTL region was considered to be a “candidate region.” A total of four local eQTL regions and four distant eQTL regions were finally obtained (Fig. 3d). A total of eight genes were identified, including *PbrNSC* (NAC STONE CELL PROMOTING FACTOR), a regulator located on the distant eQTL regions of both lignin and cellulose genes.

Our eQTL mapping indicated that PbrNSC could regulate the expression of three lignin (*Pbr4CL4*, *PbrLAC4*, and *PbrLAC5*) and three cellulose biosynthesis genes (*PbrCESA4a*, *PbrCESA7a*, and *PbrCESA8b*), as well as one transcription factor *PbrMYB169* previously reported to activate lignin biosynthesis genes during stone cell development in pear fruit [13] (Fig. 3e). We also found that the *PbrNSC* gene (Chr6:6,384,703-6,386,385) is located in an eQTL hotspot (Additional file 6). In addition, the expression patterns of *PbrNSC* and its potentially regulated genes were significantly correlated (Additional file 2: Fig. S5). Moreover, the expression of *PbrNSC* was significantly correlated with stone cell, lignin, and cellulose contents (Additional file 2: Fig. S6). Based on these results, we conclude that PbrNSC could exert regulatory impacts on lignin and cellulose biosynthesis during stone cell formation in pear.

### *PbrNSC* is involved in secondary cell wall formation

**Fig. 4:**
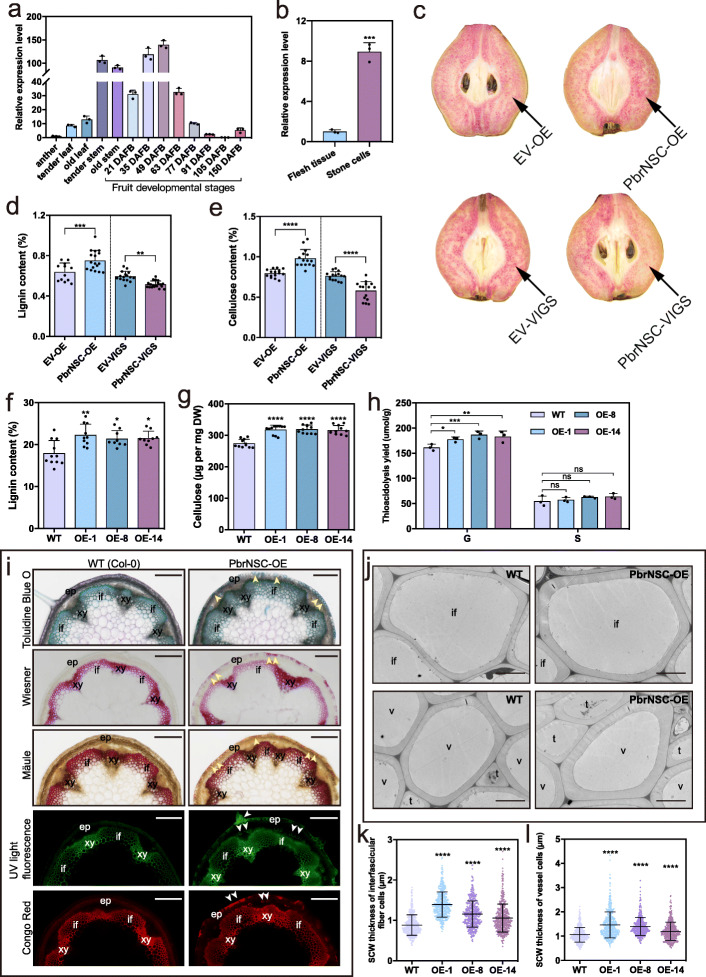
PbrNSC involved in stone cell formation. **a** Expression of *PbrNSC* in different tissues of “Dangshansuli” plants determined by qPCR. Each value is mean ± *SD* (*n* = 3 biological replicates). **b** Expression of *PbrNSC* in stone cells and flesh tissues of pear fruit at 35 DAFB. Each value is mean ± *SD* (*n* = 3 biological replicates). **c** Transient assays using *PbrNSC* overexpression and silencing constructs in “Dangshansuli” fruit at 35 DAFB. Fruit flesh was stained with Wiesner reagent, and images were taken 10 days after agro-infiltration. **d**, **e** Lignin (**d**) and cellulose (**e**) contents in the fleshy tissue around the infiltration sites. Each value is mean ± *SD* (*n* ≥ 12 biological replicates). **f**, **g** Lignin (**f**) and cellulose (**g**) contents of inflorescence stem of 8-week-old WT and transgenic plants. Each value is mean ± *SD* (*n* ≥ 3 biological replicates). **h** S- and G-lignin contents of inflorescence stems of 8-week-old WT and transgenic plants. Each value is mean ± *SD* (*n* = 3 biological replicates). **i** Cross-sections of inflorescence stems of 8-week-old plants stained with different reagents to detect lignin and cellulose deposition. The Mäule reagent specifically binds to S-lignin monomers; the Wiesner reagent binds to G-lignin monomers. Lignin autofluorescence of cross-sections was detected under UV light. Cross-sections were stained with the fluorescent dye Congo Red (for cellulose). Bar = 200 μm. xy, xylem; if, interfascicular fiber cell. A representative picture from each line is shown. **j** Transmission electron micrographs of cross-sections of interfascicular fiber and vessel cells in 8-week-old inflorescence stems. Bar = 5 μm. **k**, **l** Quantitative analysis of SCW thickness in interfascicular fiber (**k**) and vessel (**l**) cells. Three plants in each genotype and more than 20 cells in each plant were analyzed. **k** *n* = 499; **l** *n* = 474. Student’s *t*-test; *P < 0.05; **P < 0.01; ***P < 0.001; ****P < 0.0001

*PbrNSC* mRNA was abundant in stems and young fruits (21 to 63 DAFB), but at relatively low levels in anthers, leaves, and older fruits (after 77 DAFB); its transcript levels were also significantly higher in the isolated stone cells than in the flesh tissue (Fig. 4a, b). This expression pattern suggests the temporal and spatial overlap of *PbrNSC* expression and stone cell formation in pear fruit. To further elucidate the functional roles of *PbrNSC* in stone cell development, *PbrNSC* overexpression and silencing constructs were agro-infiltrated into “Dangshansuli” pear fruit at 35 DAFB. The expression of *PbrNSC* was confirmed at the tenth day after infiltration, at which a significant change of the lignin staining phenotype was observed at the infiltration sites (Fig. 4c; Additional file 2: Fig. S7). There is a clear association between the overexpression of *PbrNSC* and increased lignin and cellulose contents, and between silencing *PbrNSC* and decreased contents of lignin and cellulose (Fig. 4d, e). We also found that the expression of some known SCW biosynthesis genes were significantly changed at the injection sites of *PbrNSC* overexpression or silencing construct compared to the control (Additional file 2: Fig. S7).

We also transformed *Arabidopsis* Col-0 plants with a 35S:*PbrNSC*-*GFP* construct and showed that ectopic *PbrNSC* expression resulted in substantial increases in the expression levels of genes known to function in monolignol, cellulose, and xylan biosynthesis in T_3_ generation homozygous plants (Additional file 2: Fig. S8). The overall lignin and cellulose contents were significantly increased in the transgenic plants compared with the wild type (WT) plants at 8 weeks old (Fig. 4f, g). The lignin contained mostly G-lignin monomers in both transgenic and WT plants (Fig. 4h). Toluidine Blue O staining of the stems showed no obvious differences in the morphology of interfascicular fibers or vessel cells between *PbrNSC* overexpression (OE) and WT plants (Fig. 4i). Furthermore, Wiesner, Mäule staining and autofluorescence of lignin and Congo Red staining of cellulose showed stronger signals in the region of interfascicular fiber and xylem of *PbrNSC*-OE plants, and massive deposition of secondary walls in epidermis cells (which are normally non-sclerenchymatous); only weak staining was observed in WT plants (Fig. 4i). Thicker SCWs were present in the interfascicular fiber cells and vessel cells in *PbrNSC*-OE lines compared to the WT plants by TEM analysis (Fig. 4j–l). Together, those results suggest that *PbrNSC* functions in SCW thickening.

### LP- and WQ-box are transcriptional activation domain of PbrNSC

**Fig. 5:**
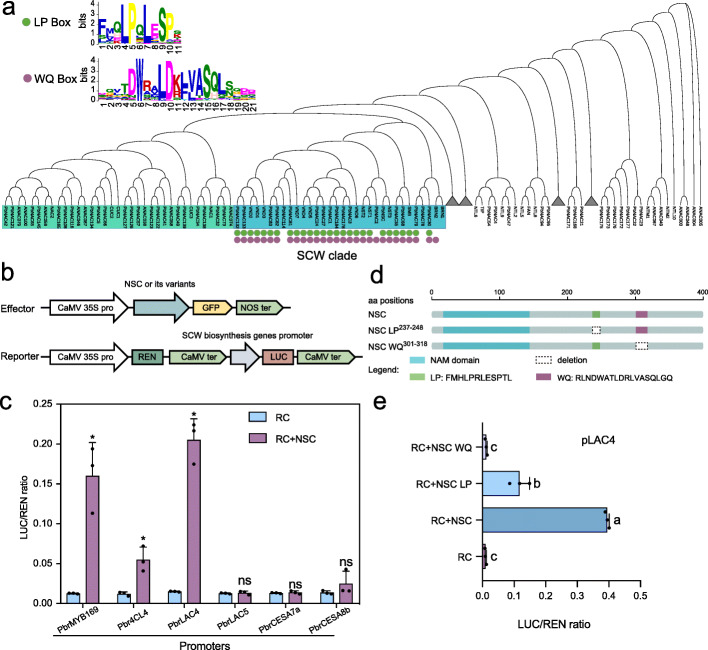
PbrNSC activates the transcription of pear SCW genes. **a** Phylogenetic tree of pear and *Arabidopsis thaliana* NAC transcription factors. Secondary cell wall biosynthesis-associated NAC transcription factors are colored in blue. A nearby subgroup is colored in green. In the phylogenetic tree, NAC proteins were indicated with green or purple circles if containing LP- or WQ-box. **b** Diagrams of the effector and reporter constructs used for the dual-luciferase assays. **c** Experimental verification of PbrNSC’s transcriptional activation function at promoters of its target genes *PbrMYB169*, *Pbr4CL4*, and *PbrLAC4*. Tobacco leaves were transfected with a luciferase reporter construct alone (RC) or together with the PbrNSC effector construct (RC + PbrNSC). Student’s *t*-test; *P < 0.05. Mean ± *SE* of three transfection repeats. **d** Schematic diagram of the primary structures of the WT PbrNSC protein and two variants. **e** PbrNSC and its various mutants on the promoter of *PbrLAC4*. Each value is mean ± *SE* of three transfection repeats. Different letters indicate significant differences between groups (*P* < 0.05, one-way ANOVA, Tukey’s HSD post hoc test)

We constructed a phylogenetic tree using the amino acid sequences of pear and *Arabidopsis* NAC transcription factors (TFs). PbrNSC was positioned near AtNST1, AtNST2, and AtNST3 which are known to function as regulators of SCW synthesis [39, 40] (Fig. 5a). In the SCW clade, *PbrNSC* was the only gene expressed abundantly during the early stage of fruit development (Additional file 2: Fig. S9). Two highly conserved motifs (an LP-box and a WQ-box) were identified in the C-terminal region of a subgroup of SCW NAC proteins including PbrNSC, VND1-7, and NST1-3 (Fig. 5a). LP- and WQ-box motifs were previously suggested to be conserved motifs present only in SCW-related NAC TFs, but their functions are unknown [41]. These motifs were not present in a nearby subgroup of NAC TFs previously shown to function in organ separation and meristem formation of *Arabidopsis* [42–45] (Fig. 5a).

We used dual-luciferase assays to examine potential interactions between PbrNSC and promoters of multiple genes associated with *PbrNSC* identified in our eQTL mapping (Fig. 3e). We found that PbrNSC interacted with promoters and activated the transcription of three genes, namely, *PbrMYB169*, *Pbr4CL4*, and *PbrLAC4* (Fig. 5b, c). The transcriptional activation region of NAC TFs is typically at the C-terminal region [46], and the LP- and WQ-box motifs of the PbrNSC are at C-terminus. We therefore speculated that these two boxes potentially function in transcriptional activation. Indeed, PbrNSC mutant without the LP-box showed 50% reductions in transcriptional activation activity and its mutant without the WP-box showed no transcriptional activation activity (Fig. 5d, e). It is worth noting that the mutants without LP- or WQ-box did not change PbrNSC subcellular localization (Additional file 2: Fig. S10).

### LP- and WQ-box of PbrNSC are responsible for activating downstream genes and affecting secondary cell wall formation

**Fig. 6:**
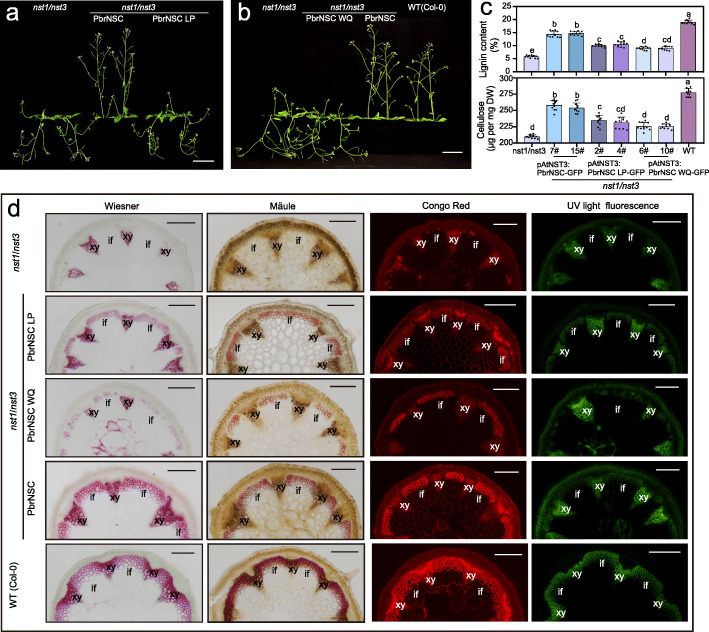
PbrNSC activates the expression of secondary cell wall formation genes. **a**, **b** Four-week-old WT *Arabidopsis* plants and *nst1*/*nst3* mutant plants with *PbrNSC*, *PbrNSC LP*, or *PbrNSC WQ* transgene. *PbrNSC LP* and *PbrNSC WQ* are variants of *PbrNSC* with deletion of the LP- and WQ-box, respectively. The transgene expression was driven by the *AtNST3* promoter (3.0 kb). Bar = 5 cm. **c** Lignin and cellulose content in inflorescence stems of 8-week-old *Arabidopsis* plants. Each value is means ± *SD* (*n* ≥ 11 biological replicates). Different letters indicate significant differences between groups (*P* < 0.05, one-way ANOVA, Tukey’s HSD post hoc test). **d** Cross-sections of inflorescence stems of 8-week-old plants stained with Wiesner, Mäule, and Congo Red for viewing G-lignin, S-lignin, and cellulose, respectively. The sections were also viewed under UV light for detecting total lignin. Bar = 200 μm. xy, xylem; if, interfascicular fiber. A representative picture from each line is shown

The *Arabidopsis* double mutant *nst1*/*nst3* has a pendent inflorescence stem phenotype because it fails to develop SCW in xylem and interfascicular fiber cells, and only develops a small amount of SCW in xylem vessel cells [39, 47]. PbrNSC is the most closely related to the AtNST1–3 proteins among *Arabidopsis* NAC proteins (Fig. 5a). To investigate the role of LP- and WQ-box during SCW synthesis, we stably transformed *nst1*/*nst3* double mutant plants with each of the three constructs wherein *PbrNSC* and its two variants without either *LP*- or *WQ*-box were driven by the *AtNST3* promoter. In T_3_ generation homozygote plants, expression of *PbrNSC* rescued the pendent inflorescence stem phenotype of *nst1*/*nst3* double mutant, but the expression of either of the two *PbrNSC* variants did not (Fig. 6a, b). This transgenic complementation with *PbrNSC* resulted in up-regulation of expression of many downstream genes known to be involved in monolignol, cellulose, and xylan biosynthesis (Additional file 2: Fig. S11). The recovery of the pendant inflorescence stem phenotype accompanied by restoration of lignin and cellulose deposition in interfascicular fibers (Fig. 6c). Stronger lignin staining and lignin autofluorescence in interfascicular fiber cells were detected in the WT and *nst1*/*nst3-PbrNSC* lines than in *nst1*/*nst3* and its transformants with either of the *PbrNSC* mutant variants (Fig. 6d). Furthermore, cellulose deposits in the interfascicular fiber cells were detected in the WT and *PbrNSC* lines, but weaker in *nst1*/*nst3* or its transgenic line containing the *PbrNSC* mutant variants (Fig. 6d). These results further support that LP- and WQ-box of PbrNSC are responsible to activate secondary cell wall-related genes to promote lignocellulose disposition.

## Discussion

Sand pear (*P. pyrifolia*) mainly cultivated in south China harbors a broad genetic diversity and phenotypic variations. It provides a useful genetic resource for studies of genomics and molecular breeding. Lignified stone cells in the sand pear fruit reduce the quality and value of this important tree fruit crop. Our goal in the current study was to understand the genetic regulation of lignin and cellulose genes in the stone cells of pear fruits.

As a complex quantitative trait controlled by multiple genes, characterizing the genetic basis of stone cell formation is challenging. However, given technological advances and significant decreases in costs, efforts through analyzing large natural populations via high-throughput sequencing have provided tremendous opportunities for exploring the genetic basis of economically important and biologically interesting traits. Although genome-wide association studies (GWAS) can help to detect genetic variation and phenotypic trait associations, it is still challenging to determine the causal genes controlling traits [48]. Gene expression links genetic variation with phenotypes, and eQTL mapping is a popular way to investigate the global genetic regulation of gene expression, including in plants like *Arabidopsis*, tomato, maize, and *Populus* [23, 25, 49–52]. Moreover, approaches that harness co-expression relationships among genes in detected regions can greatly facilitate the identification of highly plausible candidate genes related to a targeted trait. In this study, a systems genetics approach through integration of lignocellulose co-expression network and stone cell eQTL map has been successfully used to identify a regulator of lignocellulose formation in pear fruit, *PbrNSC*. In addition, there were significant differences in the stone cell (*P* < 0.05) and lignin (*P* < 0.01) contents between accessions harboring two different genotypes at one SNP position (Chr6_6,385,286) in the *PbrNSC* locus (Additional file 2: Fig. S12). This non-synonymous SNP variant, which leads to the change of Ser227 to Pro227, might affect stone cell formation through the protein function change of PbrNSC and could be used as a potential breeding marker.

Additionally, although the study here focused on pear, the predicted transcription factors in lignocellulose co-expression network based on the large panel of genetically diverse cultivars make them excellent references for the study of secondary cell wall formation in other species. Moreover, our data set could be used to explore the genetic regulation of other traits in pear fruits such as sugars, organic acids, and aroma compounds. We successfully performed a systems genetics study in a relatively large natural population for pear and then summarized the study strategy with a workflow chart, which provides a reference for comprehensive regulatory study for specific traits in other species (Additional file 2: Fig. S13). The systems genetics approach in this study can also be combined with GWAS and quantitative trait locus (QTL) analyses to facilitate the identification of candidate genes. We mapped the genes within the stone cell eQTL map against our previously reported candidate GWAS SNPs and QTLs associated with stone cell contents and were able to greatly narrow down the candidate genes (Additional file 14).

In different species, orthologues might have conserved function but embedded within a different transcriptional regulation network [53]. A previous study generated a regulatory network comprising several types of transcription factors that affect SCW formation in *Arabidopsis* [54]. The NAC TFs *AtNST1*-*3* and *AtVND1*-*7* were demonstrated as positively acting regulators controlling SCW formation in a variety of tissues [39, 40, 47, 55–58]. Complementation analysis showed that the expression of *Populus trichocarpa PtrWND*s or *Eucalyptus EgWND1* rescues the secondary wall thickening defects in fibers of the *Arabidopsis* double-knockout mutant *nst1*/*nst3* [59]. In our study, phylogenetic analysis showed that PbrNSC was closely related to AtNST3, and transgenic complementation of *nst1*/*nst3* plants with *PbrNSC* enhanced transcriptional level of SCW-related genes and unconventionally accumulated lignified SCW in *Arabidopsis* inflorescence stems. When *PbrNSC* was heterologously expressed in the *nst1*/*nst3* double mutant driving by the *Arabidopsis NST3* promoter, the pendent stem phenotype and the SCW lignification of inflorescent fibers were effectively rescued, suggesting that *PbrNSC* is functionally equivalent to *Arabidopsis NST3*. Together, these functional studies of secondary wall NAC homologs from various species suggest the similar activation of SCW deposition.

NAC proteins probably originated more than 600 million years ago [60]. Secondary wall NAC homologs are present in all taxa of land plants, including nonvascular moss plants (*Physcomitrella patens*), primitive spore-bearing vascular plants (*Selaginella moellendorffii*), gymnosperms, and angiosperms [61]. Intriguingly, *P. patens* does not have lignified secondary walls but its genome harbors eight loci with close homology to *VND*/*NST* [61, 62]. It is thus possible that vascular plants have somehow co-opted these ancestral genes and through functional diversification deployed them to regulate the formation of secondary walls. In vascular plants, most members of the secondary wall NAC subfamily have the signature LP- and WQ-box in the C-terminal of proteins, which were not discovered in nonvascular plants. The results showed that expansions of LP- and WQ-box of SCW-related NACs were essential for lignified secondary walls. It is consistent with previous conclusions that the progenitorial SCW-related NAC proteins were adapted for the regulation of SCW deposition in advanced vascular plants, mainly through the acquisition of C-terminal activation motifs [63].

## Conclusions

We used a systems genetics approach to elucidate molecular regulatory mechanisms of stone cell formation in pear fruit, which negatively affects the perceived quality of fruit. We showed that PbrNSC is a regulator of stone cell formation, thus deepening our understanding of secondary cell wall regulation in tree fruit crops. Our research demonstrates the effectiveness of a systems genetic approach for detecting regulators of complex quality traits in tree fruit crops.

## Methods

### Plant materials and RNA sequencing

A total of 206 cultivars of sand pear (*Pyrus pyrifolia*) were collected in 2016, including most of the widely cultivated varieties of this species in China (Additional file 1). According to our previous study, stone cell content increases rapidly in the early stages of pear fruit development [64]. Therefore, we collected fruit samples of different pear varieties at 49 DAFB, at which stone cell synthesis was still active. At least six randomly selected fruit samples were pooled together for each of the cultivars. Additionally, three widely cultivated varieties representing high, medium, and low levels of stone cells were also sampled from trees during fruit development at 21, 35, 49, 63, 77, 91, and 133 DAFB. At 49 DAFB, the stone cell contents were 22.62, 12.72, and 7.04 (g/100 g); the lignin contents were 3.09, 1.80, and 1.30 (g/100 g); and the cellulose contents were 1.78, 1.41, and 0.52 (g/100 g), respectively. Total RNA was extracted from fruits using TRIzol reagent, and paired-end RNA-Seq libraries were constructed according to the instructions provided by Illumina and sequenced with the HiSeq™ X platform to obtain 150-bp paired-end reads.

T-DNA insertion double mutant of *nst1*/*nst3* (CS67921) was obtained from the *Arabidopsis* Information Resource. *Arabidopsis* plants were grown in a glasshouse at 22 °C under a 16-h photoperiod.

### Measurement of the stone cell, lignin, and cellulose contents in fruit flesh

Stone cell contents and lignin contents in stone cells of pear fruit at 49 DAFB were measured as described by Tao et al. [10]. The cellulose contents in stone cells of pear fruit at 49 DAFB were measured using a previously described anthrone reagent method, with some modifications [65]. The stone cell powder was obtained from fruit flesh according to Tao et al. [10]. We weighed 0.2 g of stone cell powder in a beaker, placed the beaker in a cold-water bath, added 60 ml of 60% H_2_SO_4_, and allowed digestion for 30 min. The digested cellulose solution was then transferred into a 100-ml volumetric flask and adjusted to an etched line with 60% H_2_SO_4_, shaken well, and filtered into a separate beaker using a Buchner funnel. The 5-ml filtrate was added into a 100-ml volumetric flask, diluted with distilled water on a cold-water bath, and shaken well. Two milliliters of the solution was then transferred to a tube to which 0.5 ml of 2% anthrone reagent and 5 ml of concentrated H_2_SO_4_ were added. The mixture was shaken well and allowed to stand for 12 min. The absorbance was then measured at 620 nm, and the cellulose content in the stone cells was determined in reference to a standard curve calculated for microcrystalline cellulose (Macklin). For each trait, three independent measurements were conducted.

### RNA-seq read mapping and expression profiling

For each library, read quality was evaluated using the FastQC software (http://www.bioinformatics.babraham.ac.uk/projects/fastqc). To obtain high-quality reads, the sequencing adapters and low-quality bases were removed from the raw RNA-seq data using Trimmomatic [66] with default parameters. The cleaned paired-end reads were aligned to the pear reference genome [1] using HISAT2 (v2.0.5) [67] with the following parameters: -min-intronlen, 20; -max-intronlen, 10000; --dta-cufflinks. All uniquely mapped reads for each sample were used to determine the genome-wide expression pattern. The fragments per kilobase per million mapped fragments (FPKM) value for each gene for each sample was calculated using Cufflinks [68].

### Identification of co-expression modules

The R package weighted gene co-expression network analysis (WGCNA) [26, 69] was applied to identify gene modules with distinct expression patterns based on the FPKM data. Genes with low FPKM (mean FPKM < 1) were filtered out [70]. The FPKM values of the remaining 22,842 genes were used in module construction. The modules were obtained using the step-by-step network construction pipeline with default settings, except that the soft-thresholding powers *β* was set to 5, and the MEDissThres used was 0.3 (Additional file 2: Fig. S14a and S14b). GO enrichment analysis was performed based on the gene annotation information from the Plant Transcription Factor Database (PlantTFDB) [71].

### SNP calling

The SNP detection followed the best practices pipeline of GATK (v3.7) [72] for RNA-seq data (http://gatkforums.broadinstitute.org/wdl/discussion/3891/calling-variants-in-rnaseq). Firstly, cleaned reads were aligned to the pear reference genome using STAR aligner [73], and duplicated reads in the resulting alignment BAM files were marked using Picard Tools (http://picard.sourceforge.net). Secondly, the spliced mapped reads were filtered out using SplitNCigarReads and the qualities of all good alignments were reassigned to the default value of 60 using ReassignOneMappingQuality. Thirdly, SNPs were called based on the minimum phred-scaled confidence threshold 20 (-stand_call_conf >20) using the GATK tools HaplotypeCaller and then filtered with the following requirement: Fisher Strand values (FS) < 30.0 and quality by depth values (QD) > 2.0 using the GATK tools VariantFiltration.

### Phylogenetic and population structure analyses

A total of 150,880 SNPs with a minor allele frequency > 5% and a missing rate < 20% at fourfold degenerate sites corresponding to neutral or near-neutral variants were extracted for phylogenetic and population structure analyses. The maximum-likelihood (ML) tree with 1000 bootstraps was constructed using IQ-TREE (v2.1.4) [74] with the substitution model “PMB+F+R10.” Population structure was investigated using the program Admixture (v1.3.0) [75]. In addition, principal component analysis (PCA) using the whole SNPs identified from RNA-seq was performed with EIGENSOFT (v6.0.1) [76].

### eQTL mapping

Only genes with a median FPKM level > 0 among the cultivars were defined as expressed genes for eQTL mapping [49]. The expression level of each gene was normalized to follow a normal distribution using the “qqnorm” function in R (http://www.r-project.org). Previous reports have shown that gene expression levels can be substantially affected by non-genetic environmental and technical factors, as well as by unknown confounders [77, 78]. To eliminate hidden confounders, the Probabilistic Estimation of Expression Residuals (PEER) method was employed [79]. To maximize the sensitivity of the eQTL determination process, 20 PEER factors capturing ~ 55.0% of the total variance in gene expression were included. A total of 974,404 SNPs (minor allele frequency > 5% and missing rate < 20%) for the 206 accessions generated from RNA-seq reads were used for eQTL mapping. Associations for SNP-gene pairs were carried out using the linear regression mode of the Matrix eQTL Package [80], with the following covariates: quantile normalized expression matrices, the 20 expression PEER factors, and the first five genotyping principal components. To reduce false-positive associations between SNPs and gene expression, a rigorous threshold for the *P* value (*P* < 1.99 × 10^−12^) was produced by controlling the Bonferroni test criterion at *α* = 0.05. eQTLs were classified as local or distant eQTLs. We calculated the intergenic distance of pairwise adjacent genes and found a rapid decrease of distance at 20 kb with 90.0% pairwise genes (Additional file 2: Fig. S3). Therefore, if the SNPs were within 20 kb of the transcriptional start site or the end of a gene, it was considered a local eQTL, otherwise as a distant eQTL [23].

Distant eQTL hotspots are defined as genomic regions that control the expression of many genes. To identify potential distant eQTL hotspots, a permutation test was used to determine the statistical significance of deviation of the observed eQTL distribution from the expected uniform distribution [23, 51]. In the permutation test, we randomly assigned all distant eQTLs into 1-Mb windows in the genome and counted the number of eQTLs in each window. After 1000 permutation tests, the cut-off number (*P* ≤ 0.01) for eQTLs/Mb by chance alone would be 2140, and genome regions harboring more than 2140 eQTLs were identified as eQTL hotspots.

### Lignocellulose co-expression network

Lignin- and cellulose-related structural genes were retrieved from *Arabidopsis* [81–83] and *Eucalyptus grandis* [84]. The corresponding homologs of pear were identified by BLASTP search of these sequences against all pear protein sequences. The potential proteins were then submitted to InterProScan (http://www.ebi.ac.uk/Tools/pfa/iprscan) and SMART (http://smart.emblheidelberg.de) to confirm the conserved domains. Protein sequences containing complete domain were retained for the downstream analyses. In addition, transcription factor (TF) prediction was performed based on the Plant Transcription Factor Database (PlantTFDB) [71]. Lignin- and cellulose-related structural genes and predicted TFs in stone cell modules were used for the lignocellulose co-expression network construction.

We constructed the lignocellulose co-expression network through the following steps: First, we merged the gene expression matrices of 206 pear cultivars and 7 different developmental stages in three cultivars (Additional file 15). Then, we calculated the adjacencies using the function “adjacency” in WGCNA with a best soft power of 9. To minimize the effects of noise and spurious associations, the adjacencies were transformed into Topological Overlap Matrix (TOM) using the function “TOMsimilarity.” Moreover, the co-expression relationships including lignin and cellulose structural genes and TFs were obtained from TOM with a weight threshold of 0.01. The lignocellulose co-expression networks comprising the credible connections were visualized using Cytoscape v3.6.0 [85].

Hub genes were defined as those having high numbers of overall edges (i.e., connections). We determined the threshold number of edge via the permutation test (*P* value ≤0.01). In the permutation, each of the 2928 edges was randomly assigned into two nodes in the co-expression network, and the number of edges was then counted in each node. After 1000 permutation tests, the cut-off number (*P* value ≤0.01) for edges of the node by chance alone would be 57, and nodes with edges greater than or equal to 57 were defined as hub nodes (i.e., genes).

### Transient transformation of pear fruit flesh

To transiently overexpress *PbrNSC*, the full-length coding sequence of *PbrNSC* was fused in frame to the N-terminus of GFP under the control of the CaMV 35S promoter in the binary vector pCAMBIA1302 (p1302) to form the fusion vector 35S: *PbrNSC*-GFP. For TRV virus-induced gene silencing (VIGS), the partial coding sequences of *PbrNSC* (650–982 bp) were amplified and inserted into the vector TRV2. The *PbrNSC* overexpression and silencing constructs were transferred into GV3101 cells that were cultured in Luria-Bertani (LB) medium at 28 °C, with shaking at 200 r.p.m., for 1 day. Then, these cells were centrifuged, re-suspended in an infiltration buffer (10 mM MgCl_2_; 200 μM acetosyringone; 10 mM MES, pH 5.5) to a final concentration of *OD*_600_ 0.9–1.0 and maintained at 22 °C for 6 h. The cells were infiltrated into “Dangshansuli” fruit flesh at 35 DAFB using needleless syringes. Six fruits were injected for each construct in an experiment that was repeated three times. The transformed fruit was placed in the dark at 22 °C overnight and then incubated in a growth chamber at 22 °C under 16-h photoperiod for 10 days before being examined and imaged.

### Gene expression analysis by qRT–PCR

cDNA used for quantitative reverse transcription–PCR (qRT–PCR) analysis was synthesized using one-step genomic DNA removal and a cDNA synthesis kit (Transgen, China). qRT–PCR was performed using the LightCycler 480 SYBR GREEN Master system (Roche, USA). Primers were synthesized by Sangon Biotech Company (China) and are listed in Additional file 16. The prefixes of the pear genes (*Pbr-*) were named based on the reference genome *Pyrus bretschneideri* “Dangshansuli.” *PbrGAPDH* and *Atactin*/*AtEF1α* were used as reference genes for pear and *Arabidopsis*, respectively. Relative expression levels of each gene were calculated using the 2^−ΔΔCp^ algorithm.

### Lignin and cellulose analysis

The basal 10 cm of the main stem of 8-week-old *Arabidopsis* plants was chopped into 2-mm pieces to measure acetyl bromide-soluble lignin and cellulose content using standard procedures [86, 87]. The lignin composition was analyzed by thioacidolysis, as previously described [88]. The lignin-derived thioacidolysis monomers were identified by GC-MS, and the mean value was obtained from three independent experiments using pooled stem tissues of five plants.

### Histological analysis

Cross-sections (100-μm thickness) of inflorescence stems were cut with a Microtome (Leica VT1000S) (Leica Mikrosysteme, Germany). The sections were stained with Toluidine Blue O, Mäule, and Wiesner reagent separately and then observed using a Nikon Ni-U microscope (Nikon, Japan) [89]. The lignin autofluorescence was visualized under UV light (excitation at 355/25) by a Nikon Ti-E microscope (Nikon, Japan). For cellulose visualization, the sections were stained with Congo Red stains and visualized with emission at 470/40 nm and excitation at 525/50 nm [89]. Laser intensity, pinhole, and photomultiplier gain settings were kept constant between samples to obtain comparable images.

### Electron microscopy

TEM analysis was carried out using the stem samples fixed in a fixative [2.5% (v/v) glutaraldehyde, 4% (v/v) formaldehyde, and 50 mM sodium phosphate (pH 7.2)] and a method previously described [90]. The secondary wall thickness was measured on the TEM images of interfascicular fiber and vessel cells using the ImageJ software. Three plants in each genotype and more than 20 cells in each plant were analyzed.

### Conserved motifs and protein secondary structure analysis

MEME was used to identify conserved motifs with default parameters [91]. Amino acid sequences of NAC TFs of pear and *Arabidopsis* were downloaded from previous studies [92, 93]. FIMO [94] was used to find the LP-box (F[ML]QLPQLESP[KS]) and the WQ-box (DQ[VL]TDWRALD[KR][LF][VL]AS[QH]L[SN]Q[DE]D) from the amino acid sequences of the NAC TFs of pear and *Arabidopsis*. Matches were filtered at *P* value < 1.0 × 10^−4^.

### Dual-luciferase reporter assays

Dual-luciferase assay was carried out according to a previous report [95]. *Agrobacterium* containing the effector vector or report vector were re-suspended separately with infiltration buffer (10 mM MgCl_2_; 200 μM acetosyringone; 10 mM MES, pH 5.5) and mixed in a ratio of 9:1 to a final concentration of *OD*_600_ 0.9–1.0. Leaves of 2-week-old *N. benthamiana* plants were infiltrated with the mixed bacterial cultures using needleless syringes. Three days after infiltration, firefly luciferase (LUC) and renilla luciferase (REN) were assayed using dual-luciferase assay reagents (Promega, USA).

## Supplementary Information

**Additional file 1.** Summary of the 206 pear accessions.

**Additional file 2: Fig. S1** Gene coverage and the SNP location in the pear genome. (a) The percentage of genes with different levels of coverage (divided into 10 frequency categories) in the RNA-seq dataset. Gene coverage was here calculated as the ratio of the gene region with covered reads to the total gene length. (b) The number of SNPs in each region: Upstream refers to the area within 3 kb upstream of the start codon and downstream refers to the area within 3 kb of the stop codon. **Fig. S2** Phylogenetic tree and population structure of 206 sand pears. (a) Phylogenetic tree of sand pears. (b) Population structure of sand pears (K = 2). (c) Principal component analysis (PCA) of sand pears. Clade I in red contains accessions from China and Clade II in blue contains accessions from Japan and Korea. **Fig. S3** The distance distribution of the pairwise genes. The physical distances separating 90% of the pairwise genes were less than 20 kb; this was the distance used to define local vs. distant eQTLs. **Fig. S4** The number of transcription factors involved in the co-expression networks built for the lignin and cellulose biosynthesis. **Fig. S5** Heatmap of Pearson correlation coefficients (PPC) between the expression of *PbrNSC* and its potentially regulated genes. Numbers within the heatmap represent correlation coefficients (*r* values) and *P* values (in parentheses). The color scale indicates *r* values. **Fig. S6** Correlation between the expression of *PbrNSC* and the contents (g/100 g fruit flesh fresh weight) of stone cells, lignin and cellulose in 206 pear cultivars. **Fig. S7** Relative expression level of *PbrNSC* and genes encoding enzymes involved in secondary cell wall biosynthesis in the fleshy tissue infiltration sites in Fig. 4c. Each value is mean ± SD (n = 3 biological replicates). **Fig. S8** Expression level of secondary cell wall biosynthesis genes in inflorescence stems of four-week-old T_3_ generation transgenic plants. **Fig. S9** Expression profiles of NAC transcription factors of SCW clade in pear. Expression levels of seven different developmental stages in sand pear ‘Rongshan’ were included: 21 DAFB (S1), 35 DAFB (S2), 49 DAFB (S3), 63 DAFB (S4), 77 DAFB (S5), 91 DAFB (S6), and 134 DAFB (S7). **Fig. S10** Subcellular localization of PbrNSC, PbrNSC WQ and PbrNSC LP in root of transgenic *Arabidopsis* plants. **Fig. S11** Expression level of secondary cell wall biosynthesis genes in inflorescence stems of four-week-old Col-0 WT, *nst1*/*nst3* mutant and various complemented transgenic plants. **Fig. S12** Phenotypic divergence (*t*-test) of stone cell and lignin contents between accessions harboring the two different genotypes based on the significantly associated SNP (Chr6_6,385,286) in *PbrNSC*. **Fig. S13** Layout of the study. **Fig. S14** The criteria used in WGCNA analysis. (a) Clustering dendrogram of fruit flesh samples from 206 cultivars based on their Euclidean distance. The red line represents a threshold used to remove obvious outliers. (b) Analysis of network topology for various soft thresholds. The best value for this dataset was 5.

**Additional file 3.** The number of genes with different coverage in each accession.

**Additional file 4.** GO enrichment for modules related to stone cell content (q < 0.05).

**Additional file 5.** Number of SNPs in each region.

**Additional file 6. **List of identified distant eQTL hotspots (*P* value < 0.01).

**Additional file 7.** The reported local eQTL for enzyme genes in sugar, organic acids, aroma metabolism pathway.

**Additional file 8.** The reported distant eQTL for enzyme genes in sugar, organic acids, aroma metabolism pathway.

**Additional file 9.** The reported local eQTL for genes in stone cell modules.

**Additional file 10.** The reported distant eQTL for genes in stone cell modules.

**Additional file 11.** Lignin and cellulose gene families.

**Additional file 12.** Detailed information of all the genes in the co-expression network of lignin and cellulose.

**Additional file 13.** The genes connections in the co-expression network of lignin and cellulose.

**Additional file 14.** List of selected stone cell-related genes in previously reported GWAS and QTL regions.

**Additional file 15.** Read and mapping information of three widely cultivated varieties representing high, medium, and low levels of stone cells during fruit development at 21, 35, 49, 63, 77, 91, and 133 DAFB.

**Additional file 16.** Primer list.

**Additional file 17.** Review history.

## Data Availability

RNA-seq reads of 206 pear samples have been deposited into the NCBI Sequence Read Archive (SRA) under the accession of PRJNA723405 [96]. In addition, the data are also available from the corresponding authors upon request.
