# Supplementary material for: A systems genetics approach reveals PbrNSC as a regulator of lignin and cellulose biosynthesis in stone cells of pear fruit
Source: Genome Biol. 2021 Nov 14;22:313. doi: 10.1186/s13059-021-02531-8 (PMC8590786; doi:10.1186/s13059-021-02531-8)
Supplement: Supplementary file 2 — Additional file 2: Fig. S1 Gene coverage and the SNP location in the pear genome. (a) The percentage of genes with different levels of coverage (divided into 10 frequency categories) in the RNA-seq dataset. Gene coverage was here calculated as the ratio of the gene region with covered reads to the total gene length. (b) The number of SNPs in each region: Upstream refers to the area within 3 kb upstream of the start codon and downstream refers to the area within 3 kb of the stop codon. Fig. S2 Phylogenetic tree and population structure of 206 sand pears. (a) Phylogenetic tree of sand pears. (b) Population structure of sand pears (K = 2). (c) Principal component analysis (PCA) of sand pears. Clade I in red contains accessions from China and Clade II in blue contains accessions from Japan and Korea. Fig. S3 The distance distribution of the pairwise genes. The physical distances separating 90% of the pairwise genes were less than 20 kb; this was the distance used to define local vs. distant eQTLs. Fig. S4 The number of transcription factors involved in the co-expression networks built for the lignin and cellulose biosynthesis. Fig. S5 Heatmap of Pearson correlation coefficients (PPC) between the expression of PbrNSC and its potentially regulated genes. Numbers within the heatmap represent correlation coefficients (r values) and P values (in parentheses). The color scale indicates r values. Fig. S6 Correlation between the expression of PbrNSC and the contents (g/100 g fruit flesh fresh weight) of stone cells, lignin and cellulose in 206 pear cultivars. Fig. S7 Relative expression level of PbrNSC and genes encoding enzymes involved in secondary cell wall biosynthesis in the fleshy tissue infiltration sites in Fig. 4c. Each value is mean ± SD (n = 3 biological replicates). Fig. S8 Expression level of secondary cell wall biosynthesis genes in inflorescence stems of four-week-old T3 generation transgenic plants. Fig. S9 Expression profiles of NAC transcription factors of SCW [file 13059_2021_2531_MOESM2_ESM.docx]

**Additional file 2**


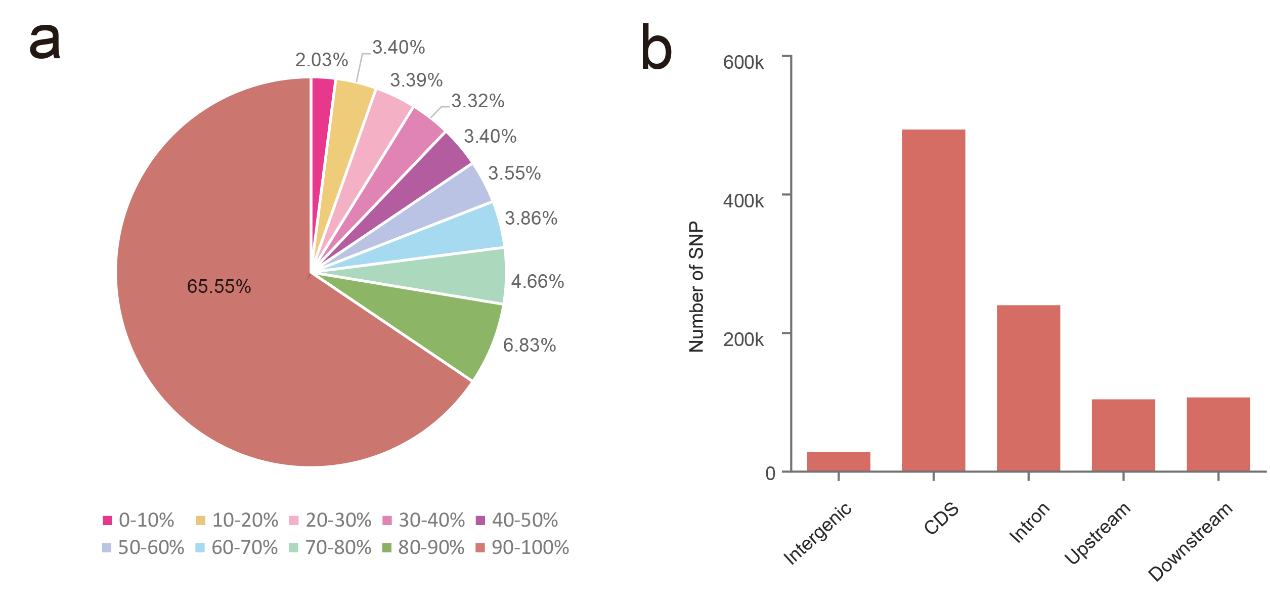


**Fig. S1 Gene coverage and the SNP location in the pear genome. (a)** The percentage of genes with different levels of coverage (divided into 10 frequency categories) in the RNA-seq dataset. Gene coverage was here calculated as the ratio of the gene region with covered reads to the total gene length. **(b)** The number of SNPs in each region: Upstream refers to the area within 3 kb upstream of the start codon and downstream refers to the area within 3 kb of the stop codon.


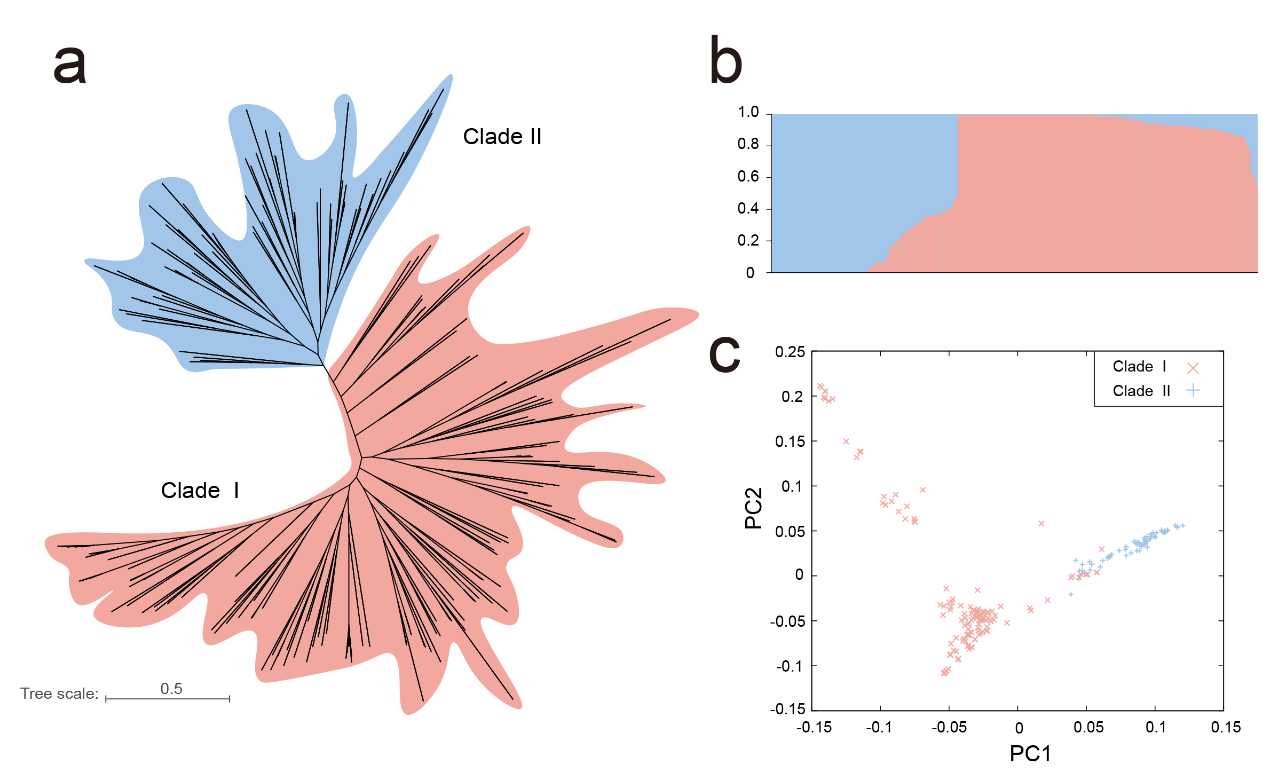


**Fig. S2 Phylogenetic tree and population structure of 206 sand pears. (a)** Phylogenetic tree of sand pears. **(b)** Population structure of sand pears (K = 2). **(c)** Principal component analysis (PCA) of sand pears. Clade I in red contains accessions from China and Clade II in blue contains accessions from Japan and Korea.


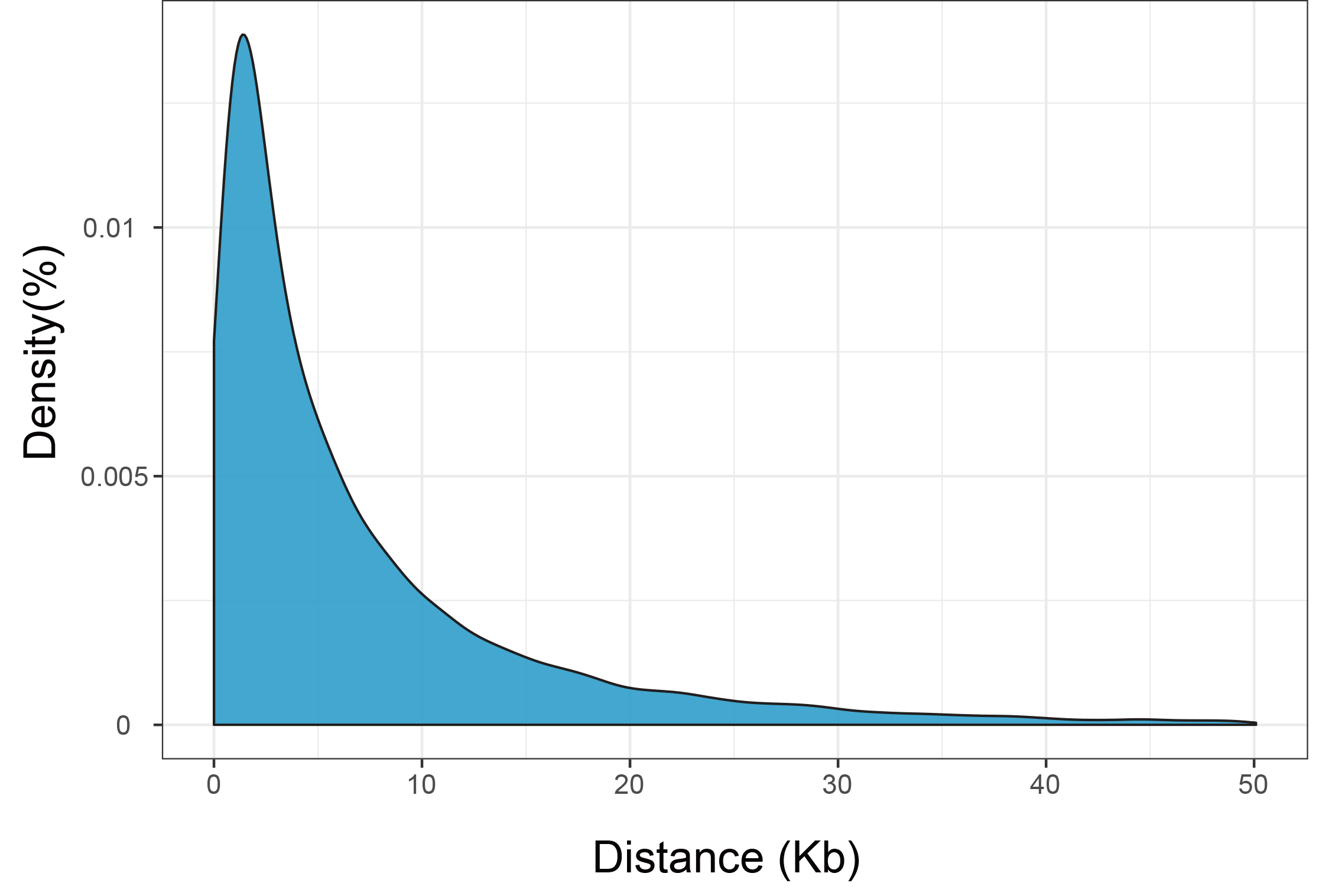


**Fig. S3 The distance distribution of the pairwise genes.** The physical distances separating 90% of the pairwise genes were less than 20 kb; this was the distance used to define local vs. distant eQTLs.


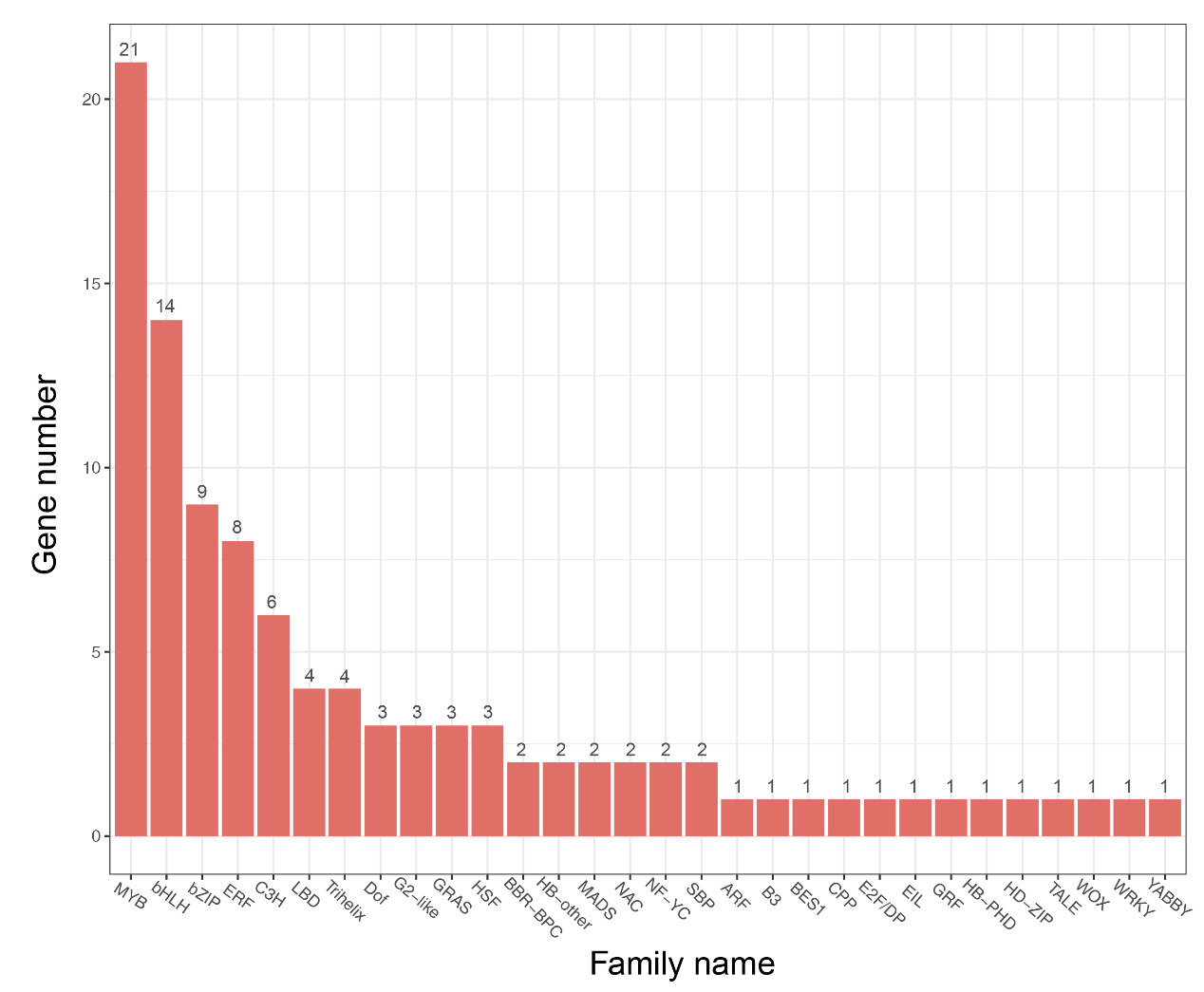


**Fig. S4** The number of transcription factors involved in the co-expression networks built for the lignin and cellulose biosynthesis.


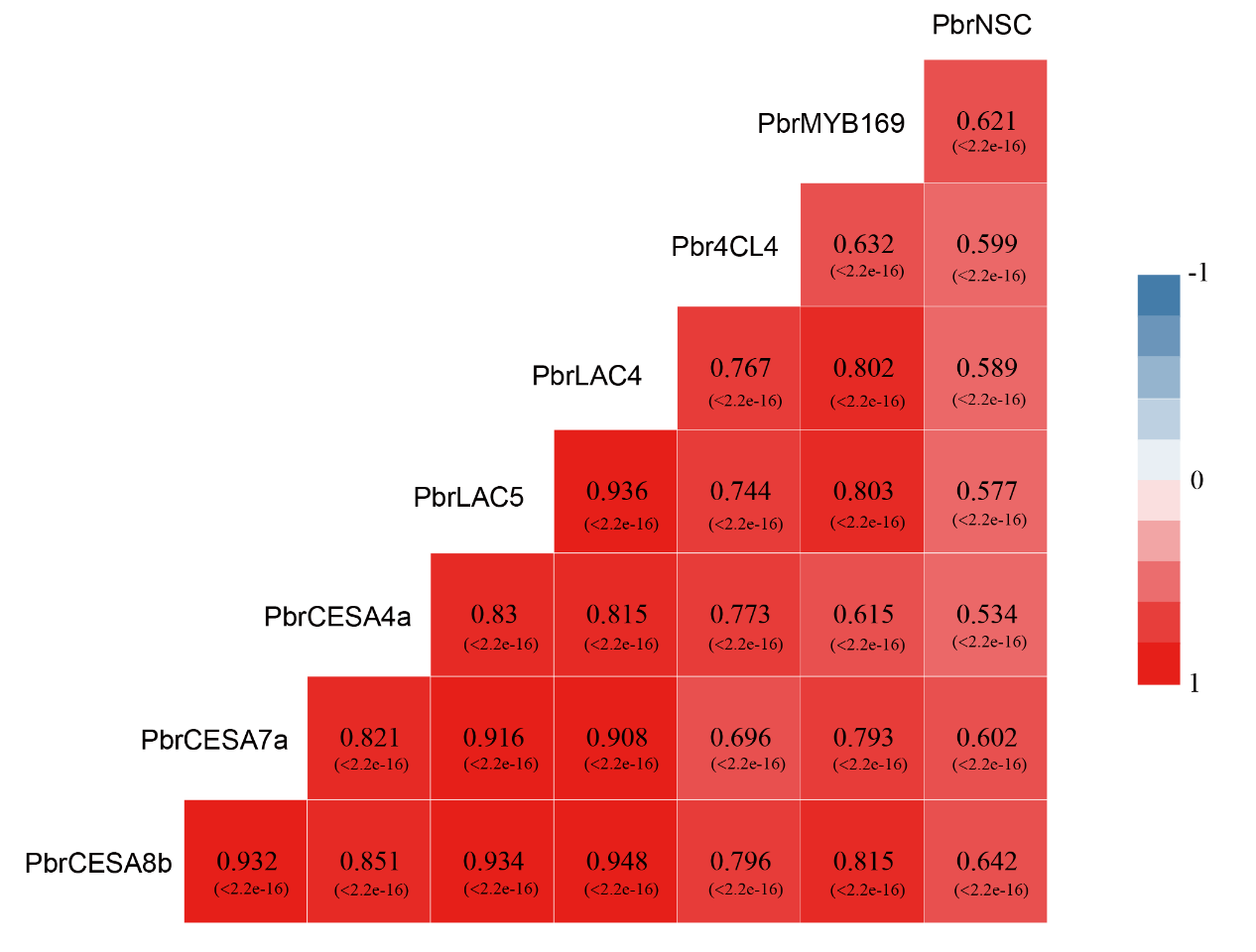


**Fig. S5** Heatmap of Pearson correlation coefficients (PPC) between the expressions of *PbrNSC* and the potential regulated genes. Numbers within the heatmap represent correlation coefficients (*r* values) and *P* values (in parentheses). The color scale indicates *r* values.


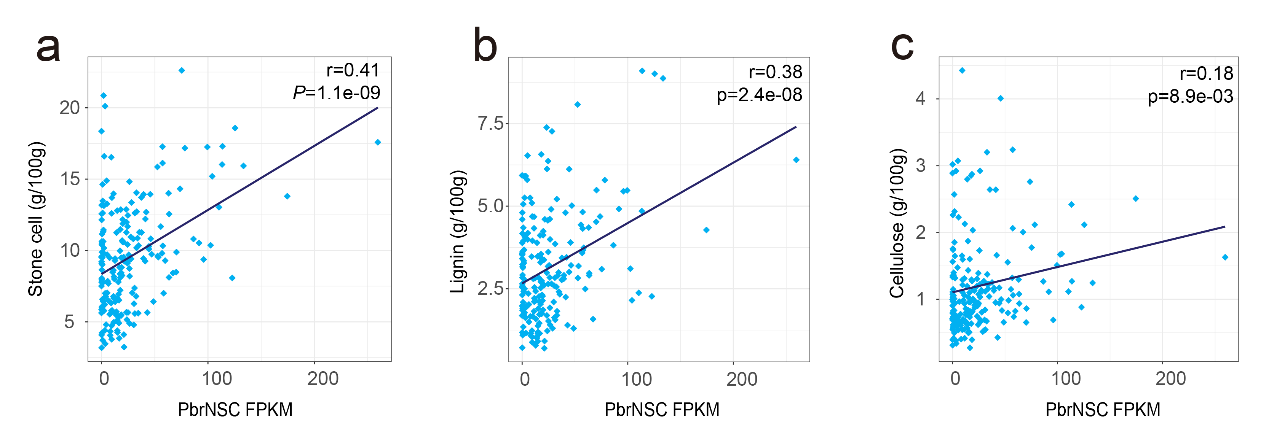


**Fig. S6** Correlation between expression of *PbrNSC* and the contents (g/100g fruit flesh fresh weight) of stone cells, lignin and cellulose in 206 pear cultivars.


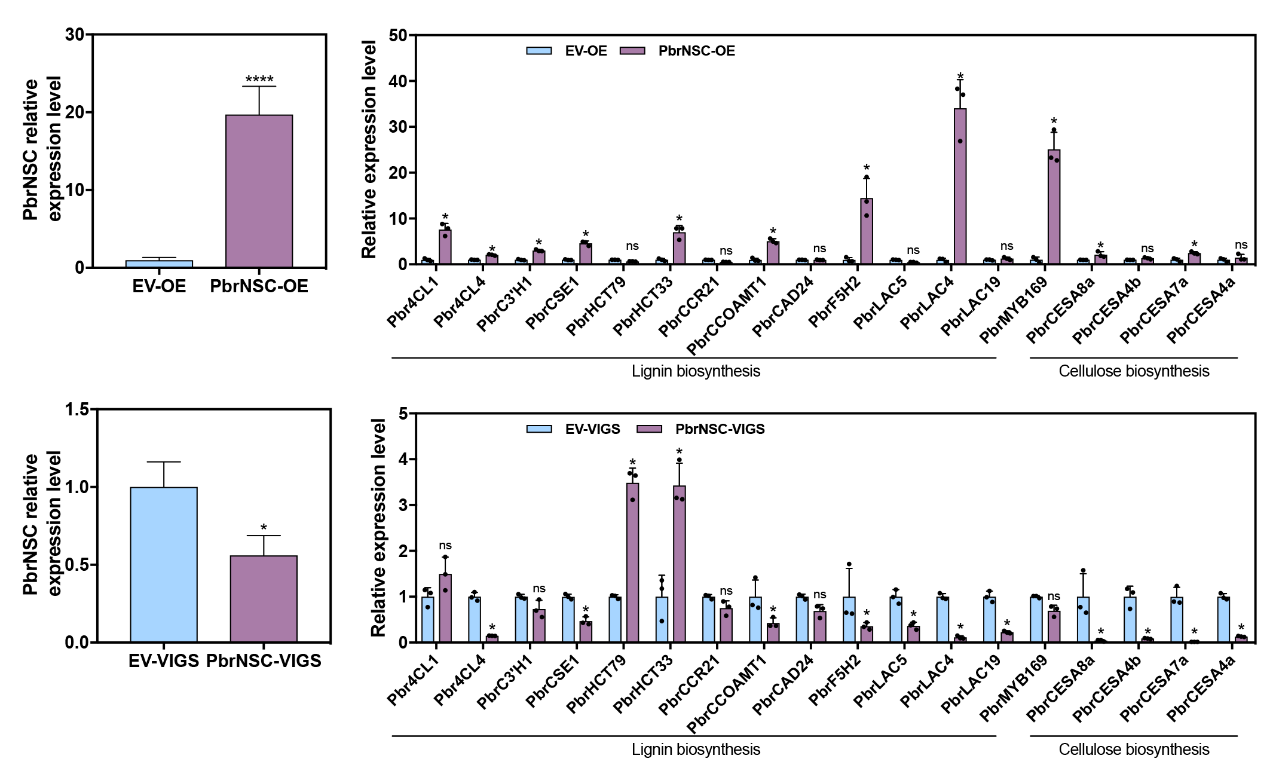
**Fig. S7** Relative expression level of *PbrNSC* and genes encoding enzymes involved in secondary cell wall biosynthesis in the fleshy tissue infiltration sites in Fig. 4c. Each value is mean ± SD (n=3 biological replicates).


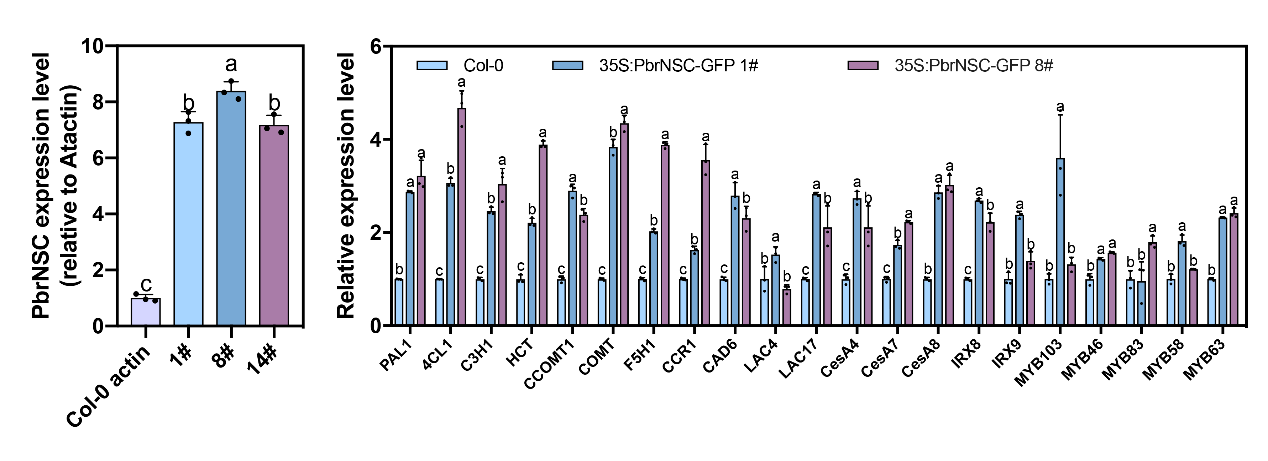


**Fig. S8** Expression level of secondary cell wall biosynthesis genes in inflorescence stems of four-week-old T3 generation transgenic plants.


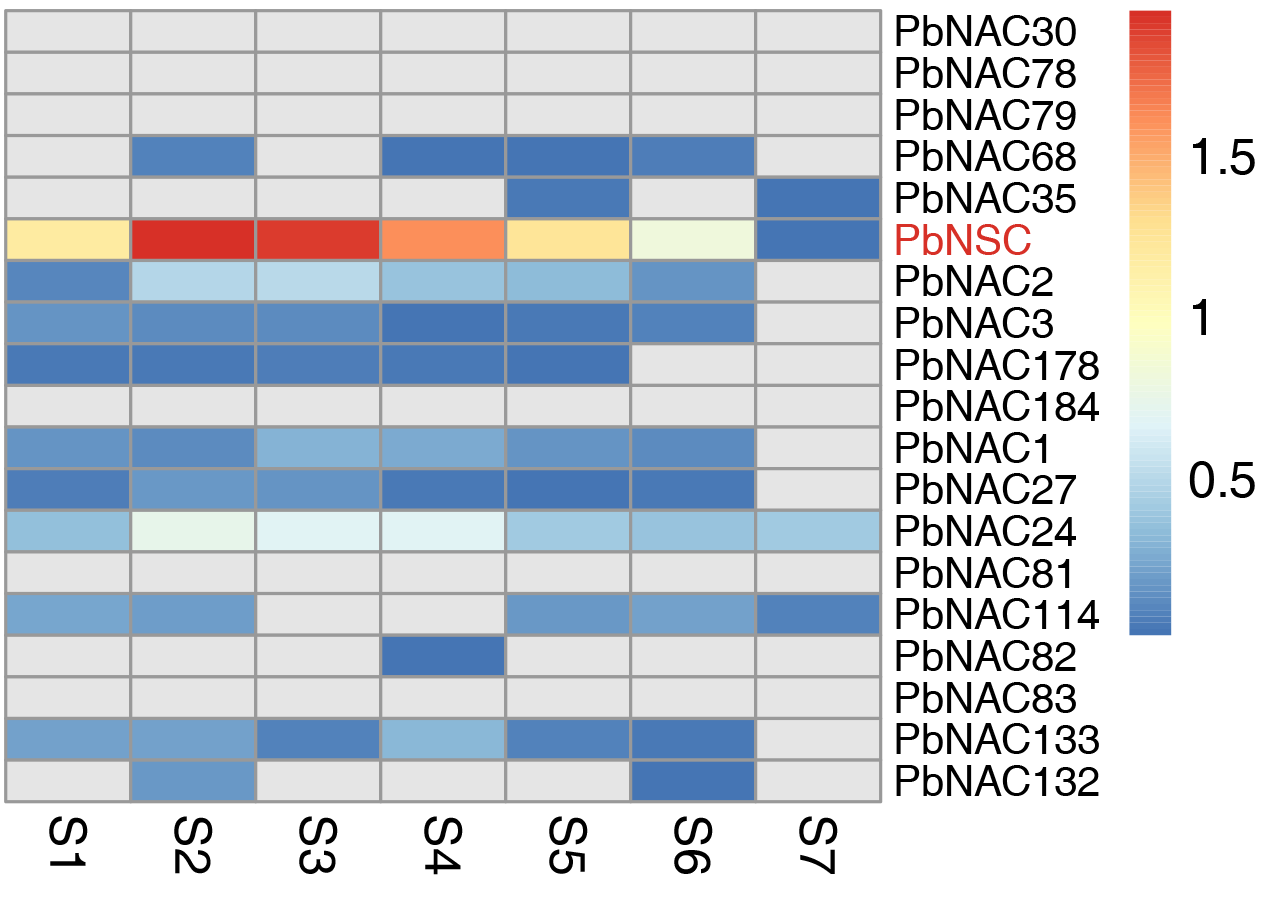


**Fig. S9 Expression profiles of NAC transcription factors of SCW clade in pear.** Expression levels of seven different developmental stages in sand pear ‘Rongshan’ were included: 21 DAFB (S1), 35 DAFB (S2), 49 DAFB (S3), 63 DAFB (S4), 77 DAFB (S5), 91 DAFB (S6), and 134 DAFB (S7).


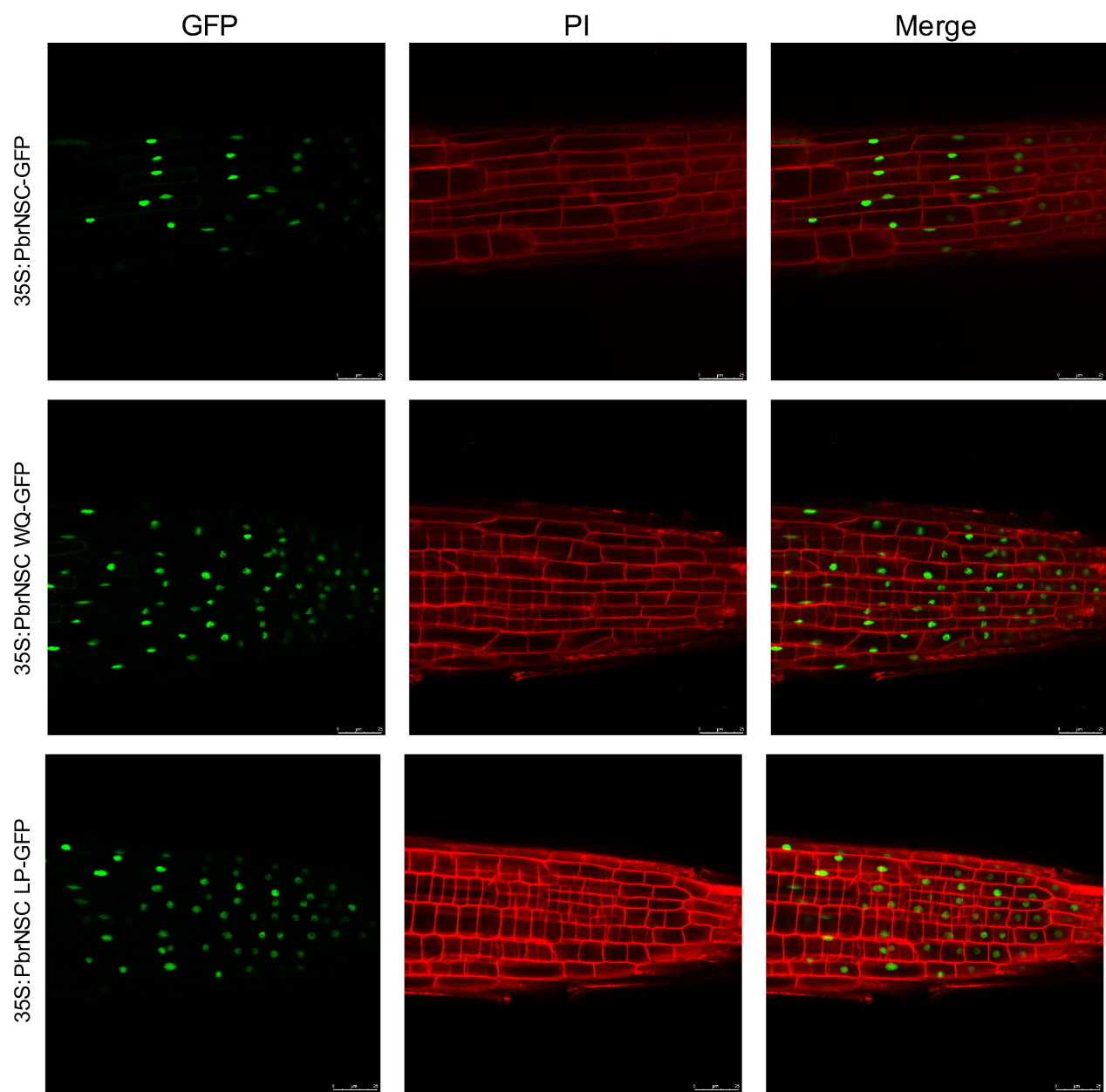


**Fig. S10** Subcellular localization of PbrNSC, PbrNSC WQ and PbrNSC LP in root of transgenic *Arabidopsis* plants.


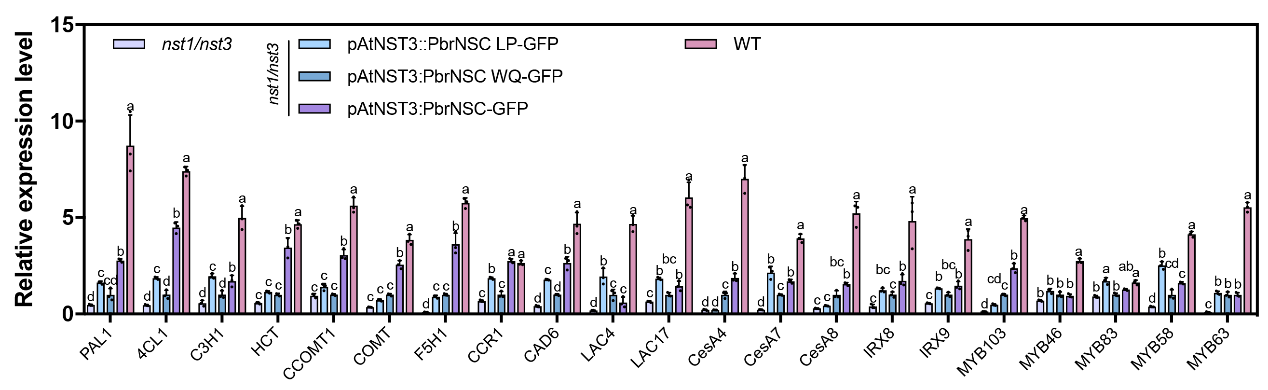


**Fig. S11** Expression level of secondary cell wall biosynthesis genes in inflorescence stems of four-week-old Col-0 WT, *nst1*/*nst3* mutant and various complemented transgenic plants.


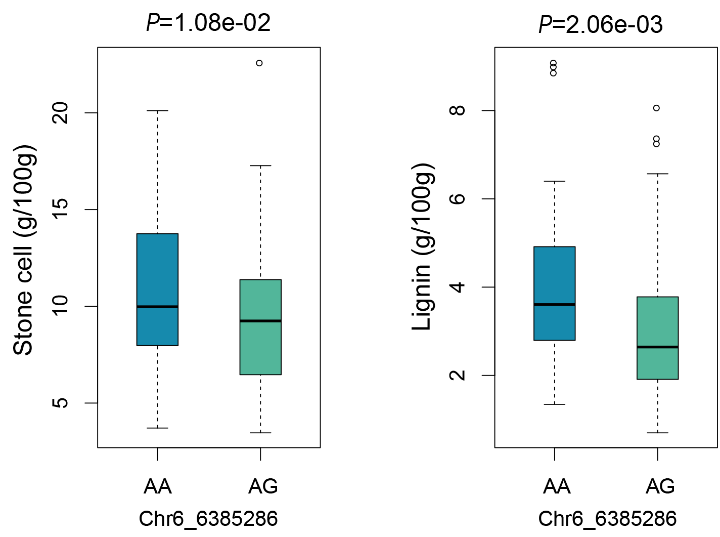
**Fig. S12** Phenotypic divergence (*t*-test) of stone cell and lignin contents between accessions harboring the two different genotypes based on the significantly associated SNP (Chr6_6,385,286) in *PbrNSC*.


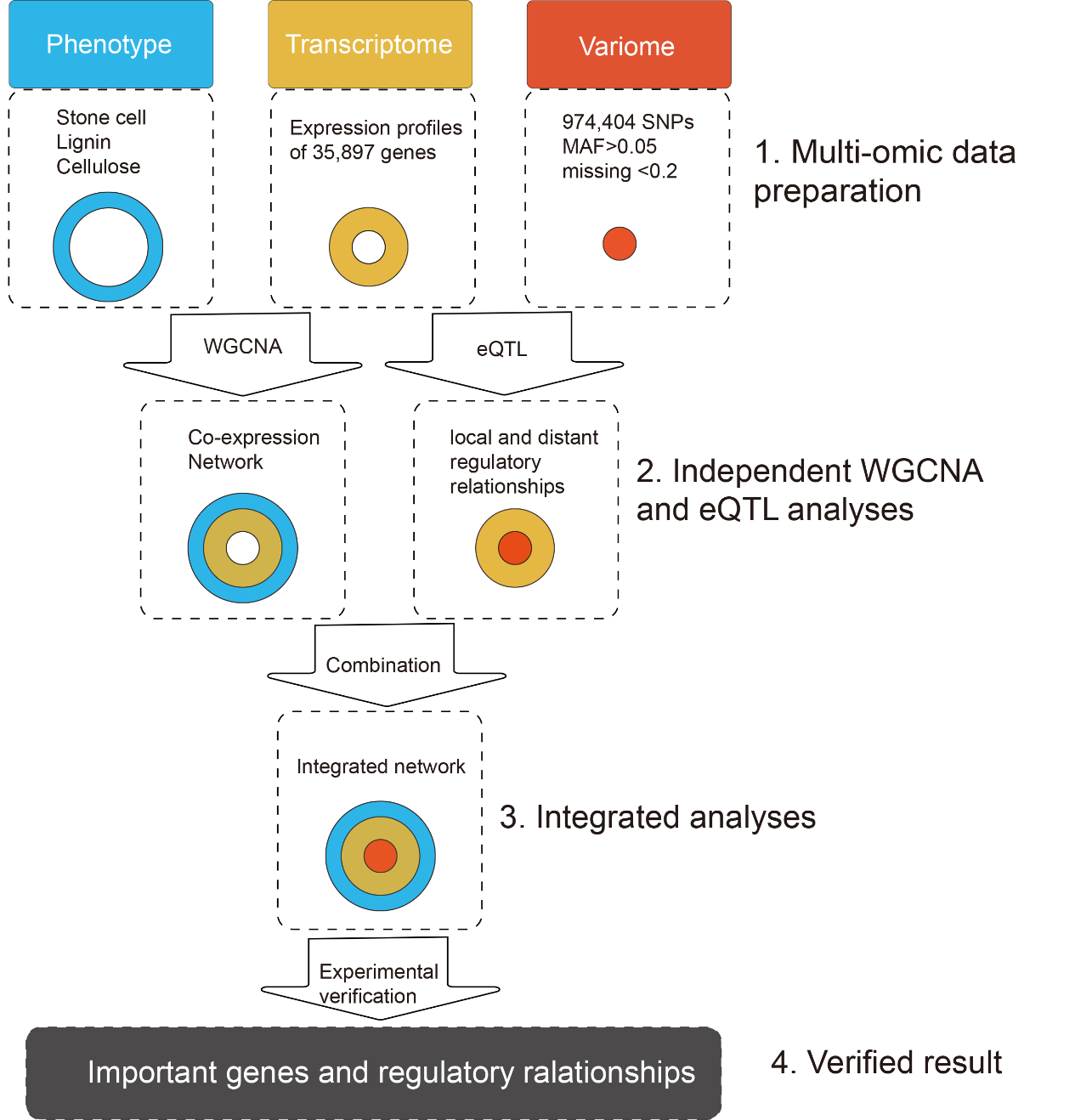


**Fig. S13** Layout of the study.


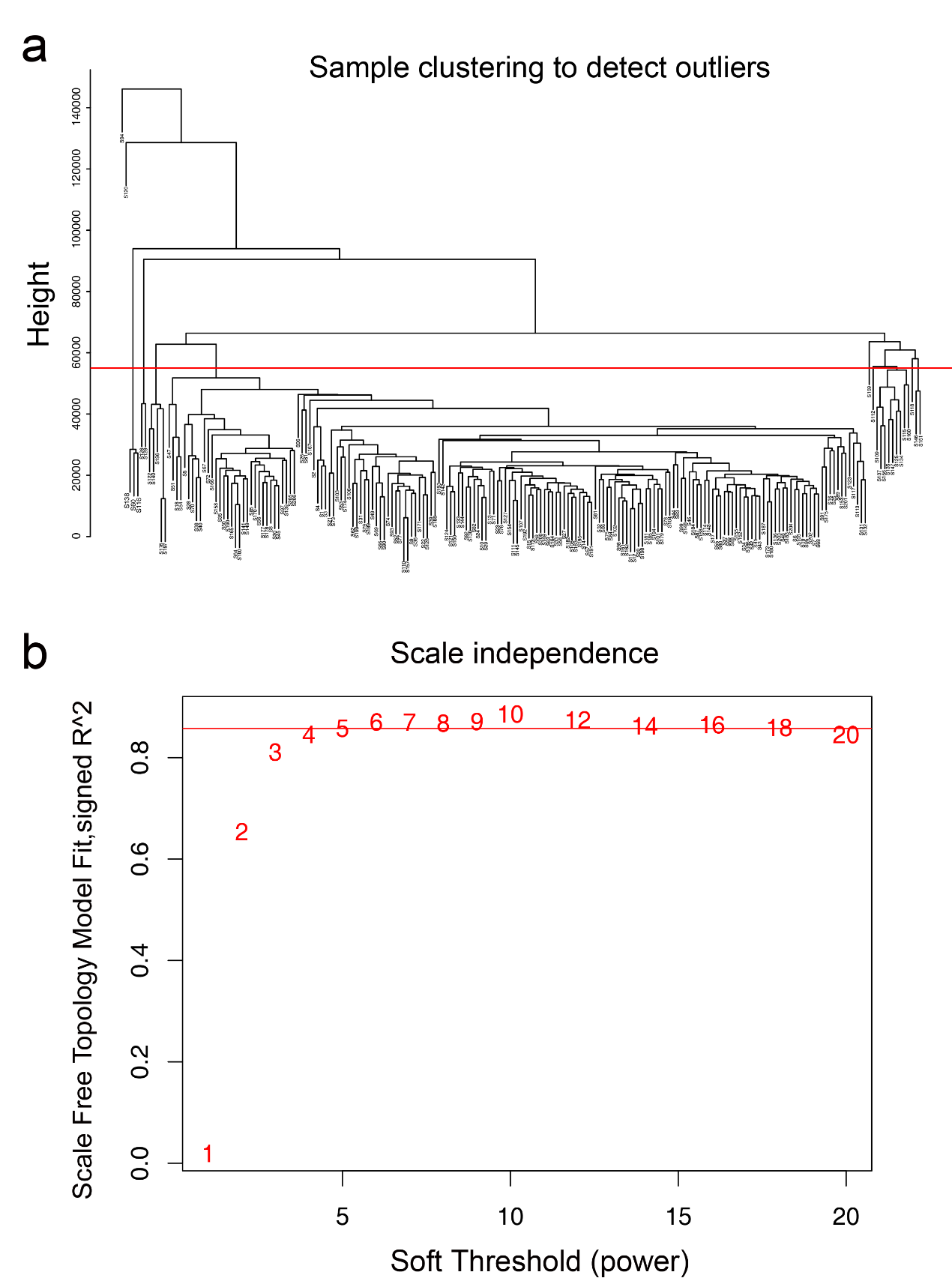


**Fig. S14 The criteria used in WGCNA analysis.** **(a)** Clustering dendrogram of fruit flesh samples from 206 cultivars based on their Euclidean distance. The red line represents a threshold used to remove obvious outliers. **(b)** Analysis of network topology for various soft thresholds. The best value for this dataset was 5.
