# Supplementary material for: A systems genetics approach reveals PbrNSC as a regulator of lignin and cellulose biosynthesis in stone cells of pear fruit
Source: Genome Biol. 2021 Nov 14;22:313. doi: 10.1186/s13059-021-02531-8 (PMC8590786; doi:10.1186/s13059-021-02531-8)
Supplement: Supplementary file 17 — Additional file 17. Review history. [file 13059_2021_2531_MOESM17_ESM.docx]

Review History

**First round of review**

**Reviewer 1**

**Are you able to assess all statistics in the manuscript, including the appropriateness of statistical tests used?**

**Yes, and I have assessed the statistics in my report.**

**Comments to author:**

In this manuscript, Wang et al. adopted an integrated strategy combing the co-expression network and eQTLs for mining the candidate genes associated with the biosynthesis of lignin and cellulose, an essential aspect in affecting the fruit texture by stone cells in the pear fruit flesh. Furthermore, the subsequent validation of the candidate gene, PbrNSC, in both the tissue of pear fruit and Arabidopsis mutants provides evidence of its functional role in regulating lignocellulose formation. It is, in principle, a good example (also a referential approach) for deciphering the fruit quality-related trait in fruit tree crops. Nevertheless, I believe some issues are needed to be further addressed, and additional analyses may need to bring more in-depth understandings before the publication in Genome Biology.

Major issues:

1. The sampled time was 49 DAFB. However, the stone cell content was stable at this time point, which suggest that the causal gene for the stone cell content was not active or did not play vital role. Why don't the authors take samples when the stone cell content changes the fast? And the stone cell content, lignin content and cellulose content were measured at the sampling time or at post-harvest? At 49 DAFB, the lignin content and cellulose content were also stable? The authors should clarify the logic for the three traits and make a clear describe for the trait they studied.

2. Line 113. The proportion of the genes detected was as high as 86.3%. However, the average mapping rate was very low (72.6%) for a diploid species in my opinion. It seems because the authors used the reference genome 'DangshanSuli', which is whole another pear species (Pyrus bretschneideri). Could the authors explain the reason of low mapping rate? In the RNA-Seq analysis, the unique mapping is required to exclude the noise from the paralogous genes or genes with high similarity. Please add more information about the quality control metrics (such as, Q20, Q30 for short reads) for raw data and the reads mapping.

3. Line 126. Although the lignin and cellulose content was positively correlated with the stone cell content, it is inappropriate to use lignin and cellulose contents as the phenotype to find the co-expression modules involved in stone cell formation. Because the lignin and cellulose content was not affected by only stone cell content. I suggest the authors to focus on the modules significantly with both stone cell content and lignin content or both stone cell content and cellulose content.

4. Line 172-175 and 414-425. The authors should re-construct the co-expression network using integrated gene expression profiles of the 206 pear cultivars and 7 different developmental stages in three cultivars. The co-expression network will be more robust when the sample size becomes larger. It is not proper to use different methods to construct one network based on different data sets. Moreover, is it possible to define the hub genes in this network and provide more possible regulators? This will offer the more useful information to find most important regulators.

Minor issues:

1. Lines 100-102. A total of 206 sand pear cultivars were used in this study. However, only providing a table is not enough for a detailed understanding on these materials. How about the additional analysis, such as their phylogenetic relationship and population structure using the SNPs identified from RNA-seq?

2. Line 128. It is reasonable to use q-value or adjusted P-value for the GO enrichment analysis. The q-value has been provided in the supplemental table. However, the authors did not use the q-value to filter the results.

3. I am confused about the criterion in define local or distant eQTLs in the main text Line 154-155 (ref 25) and in the method section Line 459-460 (ref 23). Are they refer to the same method?

4. Line 165-167. "We identified a total of lignin-related structural genes belonging to 14 families and 55 cellulose-related structural genes belonging to nine families in the pear genome". Please specify what has been done for the identification of those structural genes.

5. Line 169-170. How many structural genes were significantly associated with stone cell content?

6. Line 206, the gene PbrNSC was identified from the local eQTL regions or distant eQTL regions? Please add more details about PbrNSC gene. It is better to provide the expression patterns of PbrNSC and the potential regulated structural genes as figures.

7. Line 221-223. The authors chose to adopt the agro-infiltrated into 'Dangshansuli' pear (Pyrus bretschneideri) fruit but not a sand peach (Pyrus Pyrifolia) cultivar. Please explain the choice.

8. Line 418-419, the authors sequenced three pear accessions representing low, middle, and high levels of stone cell contents to construct a lignocellulose co-expression network. How about the lignin and cellulose content of these three accessions? Although the lignin and cellulose content positively correlated with stone cell contents as mentioned in the manuscript, the stone cell contents cannot represent the lignin and cellulose content. These are different traits. Moreover, please add more details about how to analyze these RNA-seq data.

9. The scale bar is missing in fig.1b. === Please consider the following questions: - Are the methods appropriate to the aims of the study, are they well described, and are necessary controls included? If not, please specify what is required. - Are the conclusions adequately supported by the data shown? If not, please explain - Are sufficient details provided to allow replication and comparison with related analyses that may have been performed? If not, please specify what is required. - Does the work represent a significant advance over previously published studies? - Is the paper of broad interest to others in the field, or of outstanding interest to a broad audience of biologists?

**Reviewer 2**

**Are you able to assess all statistics in the manuscript, including the appropriateness of statistical tests used?**

Yes, and I have assessed the statistics in my report.

**Comments to author:**

In this manuscript, the authors describe the discovery of a NAC family gene PbrNSC as a transcriptional regulator of lignin and cellulose biosynthesis in stone cells of pear fruit. The authors initially identified PbrNSC via the systems genetics approach with eQTL mapping and co-expression analysis, and further validated PbrNSC's function via transient expression and heterologous expression. Overall, the authors have provided solid evidence supporting the regulatory role of PbrNSC in lignin and cellulose biosynthesis in stone cells and provided new insights into the regulation of stone cell formation.

There are several concerns that need to be addressed. (1) QTL mapping: The authors collected phenotypic data of stone cell content, lignin and cellulose from a population of 206 cultivars. However, maybe I missed; I didn't see the outcomes of trait QTL mapping. This should be presented and discussed and may also need to be overlaid with the outcomes of eQTL mapping.

(2) cis- and trans-eQTL: The authors described that among the mapped eQTLs, 4,603 were local and 829,453 were distant. However, I didn't see the information about the distribution of eQTLs as intragenic vs intergenic. In addition, the information on eQTL hotspots was not presented. A relevant question: was PbrNSC an eQTL hotspot? Also, among the cultivar population, were there any SNPs in the PbrNSC gene that may potentially affect its functionality (i.e., within the LP- and WQ-box)?

(3) PbrNSC function: The authors showed that PbrNSC is likely an ortholog of AtNST3, and that PbrNSC was also expressed in other tissues (Fig. 4a). I wonder whether its natural variants (pending SNP information) also exhibit morphologic phenotypes in other tissues, given that AtNST3 is a master regulator of secondary cell wall biosynthesis and that nst1/nst3 double mutant displays pendent inflorescence stem phenotype. A general message I want to get here is that whether PBsNSC' regulatory role is limited to the stone cell (i.e. cell-specific) in pear.

(4) Functional validation: The authors presented PbrNSC overexpression result via transient infiltration. I wonder whether the authors have also attempted knockdown or knockout studies via transient infiltration, which can strengthen the conclusion on the function of PbrNSC.

**Reviewer 3**

**Are you able to assess all statistics in the manuscript, including the appropriateness of statistical tests used?**

Yes, and I have assessed the statistics in my report.

**Comments to author:**

The manuscript by Wang et al. focused on the mechanism of lignin and cellulose biosynthesis in the stone cells of pear fruit. The authors measured the fruit transcriptomes of 206 pear cultivars, based on which co-expression networks and eQTLs analysis were conducted. Nine modules for stone cell formation were identified via WGCNA and 145,400 eQTs were detected for 3,564 genes in these modules. Furthermore, a lignocellulose network consisting of 72 structural genes and correlated TFs were used to identify PbrNSC as a candidate regulator of stone cell formation. The function of PbrNSC was further verified by wet lab experiments. This study provides an ample resource to study stone cell formation in pear, and reveals PbrNSC as an important regulator. The manuscript should appeal to the readers concerning fruit biology. However, the manuscript has several major issues needed to be resolved:

1. The authors used WGCNA to identify nine gene modules related to stone cell formation (Fig. 2a), while the major finding of PbrNSC was depended on a lignocellulose co-expression network based on 72 lignin and cellulose structural genes (Fig. 3a-d). The latter lignocellulose co-expression network analysis can be conducted independent of WGCNA. The author should state more clearly about connection between these two analysis and the contribution of WGCNA analysis to the mechanistic understanding of stone cell formation. For example, what are the size of the nine WGCNA gene modules? Which of the nine modules contain the most of the structural genes? How does these modules relate to stone cells formation?

2. The analysis identified 4603 local eQTLs and 829453 distant eQTLs (Line No. 155 - 156) in pear. The ratio of local vs. distant is very different from, for example, the ratio of cis-eQTLs (152869) vs. trans-eQTLs (673) in human (GTEx Consortium, 2017) or the ratio of local (51.2%) vs. distant (48.8%) eQTLs in maize (Wang et al., 2018). The author should provide more in-depth discussion on this.

3. Figure 3d depicts combined Manhattan plots of eQTLs of genes associated with lignin and cellulose biosynthesis. Since Manhattan plot is usually draw for single gene or trait, how dose combined Manhattan plot work?

4. The authors has conducted conclusive experiments to demonstrate that PbrNSC regulates stone cell formation by transient overexpression in 'Dangshansuli' plants. However, it would be better to provide more information on this gene's relationship to the natural variation on different cultivars of pears. For example, does the cultivar have higher stone cell levels also have higher expression level of this gene? Can this gene be used as selection marker or putative target genes for future pear improvement?

Minor issues:

None.

**Authors Response**

**Point-by-point responses to the reviewers’ comments:**

Reviewer #1:

In this manuscript, Wang et al. adopted an integrated strategy combing the co-expression network and eQTLs for mining the candidate genes associated with the biosynthesis of lignin and cellulose, an essential aspect in affecting the fruit texture by stone cells in the pear fruit flesh. Furthermore, the subsequent validation of the candidate gene, PbrNSC, in both the tissue of pear fruit and Arabidopsis mutants provides evidence of its functional role in regulating lignocellulose formation. It is, in principle, a good example (also a referential approach) for deciphering the fruit quality-related trait in fruit tree crops. Nevertheless, I believe some issues are needed to be further addressed, and additional analyses may need to bring more in-depth understandings before the publication in Genome Biology.

*Reply: We thank the reviewer for the positive comments on our manuscript.*

Major issues:

1. The sampled time was 49 DAFB. However, the stone cell content was stable at this time point, which suggest that the causal gene for the stone cell content was not active or did not play vital role. Why don't the authors take samples when the stone cell content changes the fast? And the stone cell content, lignin content and cellulose content were measured at the sampling time or at post-harvest? At 49 DAFB, the lignin content and cellulose content were also stable? The authors should clarify the logic for the three traits and make a clear describe for the trait they studied.

*Reply: We’re sorry for the inaccurate description here. In our previous study we showed that the content of stone cells increased rapidly at early stages of fruit development (Xue et al., 2019) (Fig. r1 a). According to this result, stone cell formation was still active at 49 DAFB, which belong to the early developmental stages. Therefore, we chose the 49 DAFB to collect samples for the analyses. We have made this clear in the revised manuscript (Line 396-398).*

*In our study, stone cell, lignin and cellulose contents were measured at the sampling time point, 49 DAFB. This information has been added (Line 412 and 414).*

*The cellulose and lignin are main components of secondary cell walls of fruit stone cells. At 49 DAFB, biosynthesis of cellulose and lignin are active, and their contents increased in accompany with the increase of stone cell content (Fig. r1 b, c).*

*Fig. r1 Stone cell, lignin and cellulose contents. (a) Stone cell content during fruit development reported in our previous study (Xue et al., 2019). (b) Lignin content in stone cells. (c) Cellulose content in stone cells.*

*References:*

*Xue C, et al. (2019). PbrmiR397a regulates lignification during stone cell development in pear fruit. Plant Biotechnol J. 17:103-117.*

2. Line 113. The proportion of the genes detected was as high as 86.3%. However, the average mapping rate was very low (72.6%) for a diploid species in my opinion. It seems because the authors used the reference genome 'Dangshansuli', which is whole another pear species (Pyrus bretschneideri). Could the authors explain the reason of low mapping rate? In the RNA-Seq analysis, the unique mapping is required to exclude the noise from the paralogous genes or genes with high similarity. Please add more information about the quality control metrics (such as, Q20, Q30 for short reads) for raw data and the reads mapping.

*Reply: Thanks for the suggestions. Yes, we totally agree with the reviewer that the reference genome used will greatly affect the read mapping rate. To date, several pear genome sequences, including those of ‘Dangshansuli’ (Pyrus bretschneideri) (Wu et al., 2013), ‘Bartlett’ (Pyrus communis) (Linsmith et al., 2019), ‘Shanxi Duli’ (a wild accession, Pyrus betuleafolia) (Dong et al., 2020), and ‘Zhongai 1’ (an interspecific hybrid between Pyrus ussuriensis and Pyrus communis) (Ou et al., 2019), have been released. In our previous study, we found that both cultivated species Pyrus pyrifolia and Pyrus bretschneideri originated from the common wild species Pyrus pyrifolia, and were clustered into the same group that is separated from European pear, wild species and interspecific hybrid in the population analysis (Wu et al., 2018). Therefore, we chose the ‘Dangshansuli’ (Pyrus bretschneideri) genome as the reference due to ‘Dangshansuli’ has a relatively closer relationship with our sand pear accessions (Pyrus pyrifolia).*

*However, the reference genome of ‘Dangshansuli’ was assembled using the NGS data, and the genome is quite fragmented and not that complete. In addition, as a self-incompatible fruit tree with high heterozygosity (~1.02%), assembling pear genomes was challenging at that time. We think this could be the main reasons that caused relatively low mapping rates of our RNA-Seq data. Nevertheless, with the advance of sequencing technologies, we are planning to use the third-generation sequencing technologies (e.g., PacBio and ONT) to improve the reference genome of ‘Dangshansuli’ in the near future.*

*In our analysis, the average mapping rate was 72.6%, and the unique mapping rate (66%) is close to that in tomato (65%) (Zhu et al., 2018) and maize (71%) (Wang et al., 2018), which produced enough useful information for co-expression and eQTL analyses.*

*We have reanalyzed our data using only the uniquely mapped reads. The information about the quality control metrics for raw data and the read mapping has been added (Line 429-435).*

*References:*

*Dong X, et al. (2020). De novo assembly of a wild pear (Pyrus betuleafolia) genome. Plant Biotechnol J. 18:581-595.*

*Linsmith G, et al. (2019). Pseudo-chromosome-length genome assembly of a double haploid 'Bartlett' pear (Pyrus communis L.). Gigascience. 8:giz138.*

*Ou C, et al. (2019). A de novo genome assembly of the dwarfing pear rootstock 'Zhongai 1'. Sci Data. 6:281.*

*Wang X, et al. (2018). Genome-wide analysis of transcriptional variability in a large maize-teosinte population. Mol Plant. 11:443-459.*

*Wu J, et al. (2018). Diversification and independent domestication of Asian and European pears. Genome Biol. 19:77.*

*Wu J, et al. (2013). The genome of the pear (Pyrus bretschneideri Rehd.). Genome Res. 23:396-408.*

*Zhu G, et al. (2018). Rewiring of the fruit metabolome in tomato breeding. Cell. 172:249-261.*

3. Line 126. Although the lignin and cellulose content was positively correlated with the stone cell content, it is inappropriate to use lignin and cellulose contents as the phenotype to find the co-expression modules involved in stone cell formation. Because the lignin and cellulose content was not affected by only stone cell content. I suggest the authors to focus on the modules significantly with both stone cell content and lignin content or both stone cell content and cellulose content.

*Reply: We greatly appreciate this constructive suggestion. According to the suggestion, we removed modules correlated only with lignin or cellulose contents, and focused on modules correlated with both stone cell and lignin contents, or both stone cell and cellulose contents, as well as modules correlated only with stone cell content. We have added the module selection criteria in the revised manuscript (Line 125).*

4. Line 172-175 and 414-425. The authors should re-construct the co-expression network using integrated gene expression profiles of the 206 pear cultivars and 7 different developmental stages in three cultivars. The co-expression network will be more robust when the sample size becomes larger. It is not proper to use different methods to construct one network based on different data sets. Moreover, is it possible to define the hub genes in this network and provide more possible regulators? This will offer the more useful information to find most important regulators.

*Reply: Thanks for the nice suggestion. We merged the gene expression matrices of 206 pear cultivars and 7 different developmental stages in three cultivars to construct the lignocellulose co-expression network. The new lignocellulose co-expression network is shown in Fig. 3c. Compared with the previous version, the new edition of co-expression network is more refined. The detailed node-edge information is shown in Additional file 1: Table S11 and Table S12. The detailed method description for lignocellulose co-expression network construction has been added (Line 510-518).*

*We defined the hub genes as those having high numbers of overall edges (i.e., connections) (Permutation test, P value ≤ 0.01). A total of 32 hub transcription factors were identified in the lignocellulose co-expression network. Among these hub genes, the largest number of genes detected for a single TF family was the eight MYB genes. The corresponding results and methods have been added (Line 191-197, 520-526 and Additional file 1: Table S11).*

Minor issues:

1. Lines 100-102. A total of 206 sand pear cultivars were used in this study. However, only providing a table is not enough for a detailed understanding on these materials. How about the additional analysis, such as their phylogenetic relationship and population structure using the SNPs identified from RNA-seq?

*Reply: Thanks for the nice suggestion. We performed phylogenetic and population structure analyses based on the SNPs identified from RNA-seq. The phylogenetic tree suggested two distinct clades with geographic separations: Clade I included accessions from China and Clade II included accessions from Japan and Korea, which was also supported by the results of the population structure and the principal component analyses. We have added the results and methods in the revised manuscript (Line 144-148, 460-468 and Additional file 2: Fig. S2).*

2. Line 128. It is reasonable to use q-value or adjusted P-value for the GO enrichment analysis. The q-value has been provided in the supplemental table. However, the authors did not use the q-value to filter the results.

*Reply: Thanks. We have used q-value to filter the GO enrichment result in the revised manuscript (Additional file 1: Table S3).*

3. I am confused about the criterion in define local or distant eQTLs in the main text Line 154-155 (ref 25) and in the method section Line 459-460 (ref 23). Are they refer to the same method?

*Reply: We are sorry for the typo here. We used the method of ref23 (Zhu et al., 2018) to define local and distant eQTLs.*

4. Line 165-167. "We identified a total of lignin-related structural genes belonging to 14 families and 55 cellulose-related structural genes belonging to nine families in the pear genome". Please specify what has been done for the identification of those structural genes.

*Reply: We are sorry for the unclear description about the identification of structural genes. Specifically, lignin and cellulose related structural genes were retrieved from Arabidopsis (Goujon et al., 2003; Endler and Persson, 2011; Li et al., 2014) and Eucalyptus grandis (Carocha et al., 2015). The corresponding homologs of pear were identified by BLASTP search against all pear protein sequence. The potential proteins were then submitted to InterProScan (http://www.ebi.ac.uk/Tools/pfa/iprscan) and SMART (http://smart.emblheidelberg.de) to confirm the conserved domain. Protein sequences containing complete domain were retained for the downstream analyses. We have added this information in the revised manuscript (Line 500-506).*

*Reference:*

*Carocha V, et al. (2015). Genome-wide analysis of the lignin toolbox of Eucalyptus grandis. New Phytol. 206:1297-1313.*

*Endler A, et al. (2011). Cellulose synthases and synthesis in Arabidopsis. Mol Plant. 4:199-211.*

*Goujon T, et al. (2003). Genes involved in the biosynthesis of lignin precursors in Arabidopsis thaliana. Plant Physiol Bioch. 41:677-687.*

*Li S, et al. (2014). Cellulose synthesis and its regulation. Arabidopsis Book. 12:e0169.*

5. Line 169-170. How many structural genes were significantly associated with stone cell content?

*Reply: In this revision, according to the suggestion, structural genes only correlated with lignin or cellulose content were not included. Therefore, a total of 45 structural genes were identified to be associated with stone cell content (Line 174-175).*

6. Line 206, the gene PbrNSC was identified from the local eQTL regions or distant eQTL regions? Please add more details about PbrNSC gene. It is better to provide the expression patterns of PbrNSC and the potential regulated structural genes as figures.

*Reply: Thanks for the suggestion. PbrNSC was identified from the distant eQTL regions. We have added “distant” to make it clear (Line 219).*

*The expression patterns of PbrNSC and its potentially regulated structural genes were significantly correlated (Fig. r2). We have added this result in the revised manuscript (Line 227-228 and Additional file 2: Fig. S5).*

*Fig. r2 Heatmap of Pearson correlation coefficients (PPC) between the expression of PbrNSC and its potentially regulated genes. Numbers within the heatmap represent correlation coefficients (r values) and P values (in parentheses). The color scale indicates r values.*

7. Line 221-223. The authors chose to adopt the agro-infiltrated into 'Dangshansuli' pear (Pyrus bretschneideri) fruit but not a sand peach (Pyrus Pyrifolia) cultivar. Please explain the choice.

*Reply: We chose ‘Dangshansuli’ pear to do agro-infiltrated experiment for the following reasons:*

*1) According to our previous study, Pyrus bretschneideri showed similar genetic structure component with Pyrus Pyrifolia, and they derived from the same ancestor of the wild Pyrus Pyrifolia (Wu et al., 2018).*

*2) The process for fruit stone cell development in different pears, including Pyrus bretschneideri and Pyrus Pyrifolia, is similar. ‘Dangshansuli’ accumulates a high content of stone cells, which is beneficial factor for measuring stone cells after transient transformation.*

*3) We had developed a reliable transient transformation system using ‘Dangshansuli’ and successfully used this system to verify the function of PbrmiR397a and PbrSTONE in our previous studies (Xue et al., 2019; Zhang et al., 2021).*

*Reference:*

*Wu J, et al. (2018). Diversification and independent domestication of Asian and European pears. Genome Biol. 19:77.*

*Xue C, et al. (2019). PbrmiR397a regulates lignification during stone cell development in pear fruit. Plant Biotechnol J. 17:103-117.*

*Zhang MY, et al. (2021). Genome-wide association studies provide insights into the genetic determination of fruit traits of pear. Nat Commun. 12:1144.*

8. Line 418-419, the authors sequenced three pear accessions representing low, middle, and high levels of stone cell contents to construct a lignocellulose co-expression network. How about the lignin and cellulose content of these three accessions? Although the lignin and cellulose content positively correlated with stone cell contents as mentioned in the manuscript, the stone cell contents cannot represent the lignin and cellulose content. These are different traits. Moreover, please add more details about how to analyze these RNA-seq data.

*Reply: Thanks for your advice. Besides the stone cell content, we did measure lignin and cellulose contents for these three accessions. At 49 DAFB, the stone cell contents were 22.62, 12.72 and 7.04 (g/100g), the lignin contents were 3.09, 1.80 and 1.30 (g/100g), and the cellulose contents were 1.78, 1.41 and 0.52 (g/100g), respectively. We have added these results in the revised manuscript (Line 402-404).*

*Processing and analysis of this RNA-Seq dataset are same as those for the 206 accessions (Line 428-437 and 510-518).*

9. The scale bar is missing in fig.1b.

*Reply: Updated. Thanks.*

Reviewer #2:

In this manuscript, the authors describe the discovery of a NAC family gene PbrNSC as a transcriptional regulator of lignin and cellulose biosynthesis in stone cells of pear fruit. The authors initially identified PbrNSC via the systems genetics approach with eQTL mapping and co-expression analysis, and further validated PbrNSC's function via transient expression and heterologous expression. Overall, the authors have provided solid evidence supporting the regulatory role of PbrNSC in lignin and cellulose biosynthesis in stone cells and provided new insights into the regulation of stone cell formation. There are several concerns that need to be addressed.

*Reply: We thank the reviewer for the positive comments on our manuscript.*

(1) QTL mapping: The authors collected phenotypic data of stone cell content, lignin and cellulose from a population of 206 cultivars. However, maybe I missed; I didn't see the outcomes of trait QTL mapping. This should be presented and discussed and may also need to be overlaid with the outcomes of eQTL mapping.

*Reply: Generally, biparental segregating populations are used for QTL mapping. We did report QTL mapping of stone cell content in our previous studies (Wu et al., 2014; Li et al., 2019). Here, we used a natural population for the analysis, so QTL mapping was not performed. According to the suggestion, we tried the GWAS analysis of stone cell, lignin and cellulose contents using SNPs identified in the transcriptome. However, limited significant signals and candidate genes were identified (Fig. r3 a-c), which might be due to only expressed gene region were sequenced and the limited number of SNPs identified based on RNA-seq data. Therefore, we did not present the GWAS results in the manuscript.*

*In this manuscript, we mainly focus on co-expression and eQTL analyses (Fig. r3 d) to elucidate the complex regulatory network of the stone cell formation. Our analyses present a new strategy, different from QTL mapping and GWAS, to dissect complex fruit traits. In the revised manuscript, we have added the overlapping results of the eQTL mapping and our previous QTL analyses (Line 348-353).*

*Fig. r3 Manhattan plot of GWAS for stone cell (a), lignin (b) and cellulose (c) contents. (d) Manhattan plots of eQTL of a test gene (Pbr007376) in the lignocellulose co-expression network.*

*Reference:*

*Li X, et al. (2019). Comparative transcriptomic analysis provides insight into the domestication and improvement of pear (P. pyrifolia) fruit. Plant Physiol. 180:435-452.*

*Wu J, et al. (2014). High-density genetic linkage map construction and identification of fruit-related QTLs in pear using SNP and SSR markers. J Exp Bot. 65:5771-5781.*

(2) cis- and trans-eQTL: The authors described that among the mapped eQTLs, 4,603 were local and 829,453 were distant. However, I didn't see the information about the distribution of eQTLs as intragenic vs intergenic. In addition, the information on eQTL hotspots was not presented. A relevant question: was PbrNSC an eQTL hotspot? Also, among the cultivar population, were there any SNPs in the PbrNSC gene that may potentially affect its functionality (i.e., within the LP- and WQ-box)?

*Reply: Thanks for the suggestions. We have considered the genomic distribution of the eQTLs as intragenic vs intergenic. Consistent with the SNPs identified based on RNA-seq data, the majority of the eQTLs (98%) overlapped with genic regions, while the remaining 2% of eQTLs located in intergenic regions.*

*For eQTL hotspot analysis, we used 1-Mb windows to count the number of eQTLs along the genome according to the peak positions of the eQTLs, and used permutation tests to define the significance threshold. As a result, 111 eQTL hotspots were identified across the pear genome. In addition, we found that the PbrNSC gene (Chr6: 6,384,703-6,386,385) is located in an eQTL hotspot. We have added the related results and method in the revised manuscript (Line 159-161, 225-226 and 491-498).*

*To explore the potential SNPs in the eQTL regions overlapping the PbrNSC locus that might affect the function of PbrNSC, we annotated a total of 52 SNPs in this locus and found that there is no significant SNP in the PbrNSC locus that affects the expression of the PbrNSC gene, indicating that PbrNSC is not regulated by local eQTLs detected in this study. However, we did identify one SNP (Chr6_6,385,286) in the PbrNSC locus that was significantly associated with stone cell (P = 5.37e-3) and lignin contents (P = 4.89e-4). There were also significant differences in the stone cell and lignin contents between accessions with the two different genotypes at this SNP locus (Fig. r4). This non-synonymous SNP variant leads to the change of Ser227 to Pro227, but is not located in the LP- and WQ-box. We speculate that a yet unknown mechanism of this SNP might underlie its effects in the protein function but not the transcript expression in regulating stone cell formation. We have added the related discussion in the revised manuscript (Line 334-339).*

*Fig. r4 Phenotypic divergence (t-test) of stone cell and lignin contents between accessions with the two different genotypes based on the significantly associated SNP (Chr6_6,385,286) in PbrNSC.*

(3) PbrNSC function: The authors showed that PbrNSC is likely an ortholog of AtNST3, and that PbrNSC was also expressed in other tissues (Fig. 4a). I wonder whether its natural variants (pending SNP information) also exhibit morphologic phenotypes in other tissues, given that AtNST3 is a master regulator of secondary cell wall biosynthesis and that nst1/nst3 double mutant displays pendent inflorescence stem phenotype. A general message I want to get here is that whether PBsNSC' regulatory role is limited to the stone cell (i.e. cell-specific) in pear.

*Reply: We believe the regulatory role of PbrNSC is not limited to the stone cell in pear. However, further studies are needed to investigate its potential functional roles in other tissues, which we think is beyond the scope of this study that is mainly focused on the pear fruit quality and stone cell biosynthesis.*

(4) Functional validation: The authors presented PbrNSC overexpression result via transient infiltration. I wonder whether the authors have also attempted knockdown or knockout studies via transient infiltration, which can strengthen the conclusion on the function of PbrNSC.

*Reply: We greatly appreciate your suggestion. In the revised manuscript, we further verified the function of PbrNSC via transient gene silencing assay. Silencing the transcription of PbrNSC significantly repressed lignin and cellulose biosynthesis of stone cells in pear fruit. This result has been added (Line 239-248 and Fig. 4c-e).*

Reviewer #3:

The manuscript by Wang et al. focused on the mechanism of lignin and cellulose biosynthesis in the stone cells of pear fruit. The authors measured the fruit transcriptomes of 206 pear cultivars, based on which co-expression networks and eQTLs analysis were conducted. Nine modules for stone cell formation were identified via WGCNA and 145,400 eQTs were detected for 3,564 genes in these modules. Furthermore, a lignocellulose network consisting of 72 structural genes and correlated TFs were used to identify PbrNSC as a candidate regulator of stone cell formation. The function of PbrNSC was further verified by wet lab experiments. This study provides an ample resource to study stone cell formation in pear, and reveals PbrNSC as an important regulator. The manuscript should appeal to the readers concerning fruit biology.

*Reply: We thank the reviewer for the positive comments on our manuscript.*

However, the manuscript has several major issues needed to be resolved:

1. The authors used WGCNA to identify nine gene modules related to stone cell formation (Fig. 2a), while the major finding of PbrNSC was depended on a lignocellulose co-expression network based on 72 lignin and cellulose structural genes (Fig. 3a-d). The latter lignocellulose co-expression network analysis can be conducted independent of WGCNA. The author should state more clearly about connection between these two analysis and the contribution of WGCNA analysis to the mechanistic understanding of stone cell formation. For example, what are the size of the nine WGCNA gene modules? Which of the nine modules contain the most of the structural genes? How does these modules relate to stone cells formation?

*Reply: Thanks for the suggestion. We first identified stone cell modules based on WGCNA, then identified lignin and cellulose related structural genes and transcription factors in the stone cell modules. Moreover, lignocellulose co-expression network was constructed based on these lignin and cellulose related structural genes and TFs in stone cell modules. So, the co-expression network was not constructed independent of WGCNA. To make this clear, we have added the corresponding description in Results and Methods (Line 171-172 and 507-509).*

*The WGCNA analysis did contribute to the mechanistic understanding of stone cell formation. We first used WGCNA analysis to narrow down candidate genes by identifying candidate modules through module-trait associations. Then we focused on the co-expression and eQTL regulatory relationship between the genes of the stone cell modules. The number of genes in these seven modules (the results were updated based on the comments of the first reviewer) were 2219 (M14), 1518 (M11), 232 (M13), 140 (M12), 117 (M26), 77 (M25), and 80 (M10), respectively. The M13 module contains the highest number of lignin and cellulose related structural genes (18). In addition, GO analysis showed that different modules have distinct GO enrichment characteristics, and these modules may affect the formation of stone cells from different aspects. We have added the relevant information in the revised manuscript (Line 126-139, and 176-178).*

2. The analysis identified 4603 local eQTLs and 829453 distant eQTLs (Line No. 155 - 156) in pear. The ratio of local vs. distant is very different from, for example, the ratio of cis-eQTLs (152869) vs. trans-eQTLs (673) in human (GTEx Consortium, 2017) or the ratio of local (51.2%) vs. distant (48.8%) eQTLs in maize (Wang et al., 2018). The author should provide more in-depth discussion on this.

*Reply: Thanks for the suggestion. We retrieved the published eQTL articles of human researches and found that local eQTLs accounted for a higher ratio (Bryois et al., 2014; Aguet et al., 2017; Fagny et al., 2017), which is different from plants such as Arabidopsis (46.1% local, 48.1% distant, and 5.8% other eQTLs) (Keurentjes et al., 2007), maize (51.2% local vs. 48.8% distant eQTLs) (Wang et al., 2018), and tomato (2.7% local vs. 97.3% distant eQTLs) (Zhu et al., 2018). We think that this difference is mainly due to different characteristics of species and tissues (thus different biological processes). In addition, the difference in the interval definition of local eQTLs has also led to some differences of the ratios. In human studies, the local eQTL interval was defined as 1 Mb up- and down-stream of the transcriptional start site of genes, which greatly increases the number of local eQTLs compared to the ~20-30 kb interval commonly used in plants. Therefore, the above two aspects might lead to the difference in the ratios of local and distant eQTLs in different studies.*

*For plants, we found that many factors, including the association population (Li et al., 2013; Wang et al., 2018), tissue type (Fu et al., 2013; Liu et al., 2020), and methods of identifying and defining eQTLs (Fu et al., 2013; Liu et al., 2017) can also lead to the differences in ratios of local and distant eQTLs. Among these factors, we believe that the population difference is mainly responsible for the different ratios of local and distant eQTLs for pear and maize (Wang et al., 2018). Unlike the recombined inbred lines used in maize, we used a natural population in this manuscript, so more small effects (such as heterozygosity effects on gene expression) could be detected (Swanson-Wagner et al., 2009), which greatly increases the number of distant eQTLs. In addition, the difference in methods of identifying and defining eQTLs may also cause differences for the ratios. We referred to the method of the tomato study (Zhu et al., 2018) and used a fixed size interval to merge the significant SNPs into an eQTL. It is different from the maize study, which used LD to merge significant SNPs. Since natural populations were used as materials in pear and tomato (Zhu et al., 2018), and similar methods and parameters were used for the analyses, their ratios of local and distant eQTLs are similar. We don’t think an in-depth discussion on this is necessary. If the editor and the reviewer still think the discussion should be included, we will be happy to comply.*

*Reference:*

*Aguet F, et al. (2017). Genetic effects on gene expression across human tissues. Nature. 550:204-213.*

*Bryois J, et al. (2014). Cis and trans effects of human genomic variants on gene expression. PLoS Genet. 10:e1004461.*

*Fagny M, et al. (2017). Exploring regulation in tissues with eQTL networks. Proc Natl Acad Sci U S A. 114:e7841-e7850.*

*Fu J, et al. (2013). RNA sequencing reveals the complex regulatory network in the maize kernel. Nat Commun. 4:2832.*

*Keurentjes JJB, et al. (2007). Regulatory network construction in Arabidopsis by using genome-wide gene expression quantitative trait loci. Proc Natl Acad Sci U S A. 104:1708-1713.*

*Li L, et al. (2013). Mendelian and non-Mendelian regulation of gene expression in maize. PLoS Genet. 9:e1003202.*

*Liu H, et al. (2017). Distant eQTLs and non-coding sequences play critical roles in regulating gene expression and quantitative trait variation in maize. Mol Plant. 10:414-426.*

*Liu S, et al. (2020). Mapping regulatory variants controlling gene expression in drought response and tolerance in maize. Genome Biol. 21:163.*

*Swanson-Wagner RA, et al. (2009). Paternal dominance of trans-eQTL influences gene expression patterns in maize hybrids. Science. 326:1118-1120.*

*Wang X, et al. (2018). Genome-wide analysis of transcriptional variability in a large maize-teosinte population. Mol Plant. 11:443-459.*

*Zhu G, et al. (2018). Rewiring of the fruit metabolome in tomato breeding. Cell. 172:249-261.*

3. Figure 3d depicts combined Manhattan plots of eQTLs of genes associated with lignin and cellulose biosynthesis. Since Manhattan plot is usually draw for single gene or trait, how dose combined Manhattan plot work?

*Reply: This type of combined Manhattan plots is feasible and has been reported in several studies. We referred to the Figure 5 of Zhang et al. (2017), in which multiple Manhattan plots were combined and displayed in a single plot. Specifically, we combined all significant SNP signals in local and distant eQTLs. To make this clear, we have added the detailed description in the legend of Fig. 5.*

*Reference:*

*Zhang L, et al. (2017). RNA sequencing provides insights into the evolution of lettuce and the regulation of flavonoid biosynthesis. Nat Commun. 8:2264.*

4. The authors has conducted conclusive experiments to demonstrate that PbrNSC regulates stone cell formation by transient overexpression in 'Dangshansuli' plants. However, it would be better to provide more information on this gene's relationship to the natural variation on different cultivars of pears. For example, does the cultivar have higher stone cell levels also have higher expression level of this gene? Can this gene be used as selection marker or putative target genes for future pear improvement?

*Reply: Thanks for the suggestion. The expression of PbrNSC and the stone cell content are significantly correlated (r = 0.41, P = 1.1e-09), and the corresponding plot is shown in the figure below (Fig. r5 a). We have added this result in the revised manuscript (Line 228-230).*

*To find potential natural variation, we performed a single marker regression analysis according to the study of Balmant et al. (2020). We found that one SNP (Chr6_6,385,286) in the PbrNSC locus was significantly associated with stone cell (P = 5.37e-3) and lignin contents (P = 4.89e-4). There were also significant differences in the stone cell and lignin contents between accessions with the two different genotypes at this SNP locus (Fig. r5 b). Therefore, this could be used as potential breeding markers. We have added more detailed information in the discussion (Line 334-339).*

*Fig. r5 (a) Correlation of the expression of PbrNSC with stone cell content. (b) Phenotypic divergence (t-test) of stone cell and lignin contents between accessions with the two different genotypes based on the significantly associated SNP (Chr6_6,385,286) in PbrNSC.*

*Reference:*

*Balmant KM, et al. (2020). Xylem systems genetics analysis reveals a key regulator of lignin biosynthesis in Populus deltoides. Genome Res. 30:1131-1143.*

**Second round of review**

**Reviewer 1**

The authors have done an excellent job in addressing the comments. I suggest the manuscript should get accepted. Please check the threshold (P value < 0.01) for the Permutation test (Line 521-522), as the authors provided a different one (Permutation test, P value ≤ 0.01) in the response letter.

**Reviewer 2**

In the revised manuscript, the authors have addressed all the concerns I raised previously. I particularly appreciated that the authors have provided new results on PbrNSC's functional validation using VIGS silencing. I also appreciated that the authors have provided new results on PbrNSC SNP information and its association with stone cell and lignin content. Both of these new results have strengthened the conclusions made in this study and improved the quality of the manuscript. Overall, the authors have also provided thorough responses to concerns raised by other reviewers.

**Reviewer 3**

The authors have addressed my previous concerns.

**Authors Response**

**Point-by-point responses to the reviewers’ comments:**

Reviewer #1:

The authors have done an excellent job in addressing the comments. I suggest the manuscript should get accepted. Please check the threshold (P value < 0.01) for the Permutation test (Line 521-522), as the authors provided a different one (Permutation test, P value ≤ 0.01) in the response letter.

*Reply: We used the threshold of P value ≤ 0.01 for the Permutation test. The typo in the manuscript has been corrected in this revision. Thanks so much for your reminder.*
